# Genome-Wide Analysis of the Amino Acid Auxin Permease (AAAP) Gene Family and Identification of an AAAP Gene Associated with the Growth and Reproduction of the Brown Planthopper, *Nilaparvata lugens* (Stål)

**DOI:** 10.3390/insects12080746

**Published:** 2021-08-18

**Authors:** Lei Yue, Rui Pang, Hu Tian, Ziying Guan, Mingzhao Zhong, Luyao Zhao, Kai Liu

**Affiliations:** 1 Innovative Institute for Plant Health, College of Agriculture & Biology, Zhongkai University of Agriculture and Engineering, Guangzhou 510225, China; nongda-yuelei@163.com (L.Y.); gziying1995@163.com (Z.G.); chungmingchiu@163.com (M.Z.); Z23418765@163.com (L.Z.); 2 Guangdong Provincial Key Laboratory of Microbial Safety and Health, State Key Laboratory of Applied Microbiology Southern China, Institute of Microbiology, Guangdong Academy of Sciences, Guangzhou 510070, China; 3 Caofeidian Customs House, Tangshan 063200, China; tianhu310@163.com

**Keywords:** *Nilaparvata lugens*, amino acid auxin permease, phylogenetic analysis, expression pattern, growth, fecundity

## Abstract

**Simple Summary:**

Amino acids are one of the essential nutrients in organisms and play unique roles in multiple life activities. However, most insects cannot synthesize several amino acids and must acquire them from dietary sources. Diffusion of amino acids into and out of insect cells is heavily dependent on amino acid transporters. The amino acid auxin permease represents one of the most important amino acid transporter gene families in insects. However, amino acid transporters in most insects are not well understood. Here, we performed genome-wide identification of the amino acid auxin permeases in the brown planthopper (BPH), *Nilaparvata lugens*, a devastating pest that feeds on the phloem sap of rice plants. The molecular traits and evolutionary patterns of these putative amino acid auxin permeases in BPH were analyzed. An amino acid auxin permease which was predicted to regulate BPH nymphal growth and female fecundity was identified and functionally validated through RNA interference and bioassay experiments. Our results provide a basis for further functional research on the amino acid auxin permeases in BPH and suggest new ideas for the management of this pest.

**Abstract:**

Amino acids play a vital role in several biological processes in organisms and are mainly acquired through diet by most insects. The amino acid auxin permease (AAAP) transporter family is an important amino acid transporter gene family in insects for the transportation of amino acids into and out of cells across the plasma membrane. Here, we identified 21 putative AAAP family members in the genome of the brown planthopper (BPH), *Nilaparvata lugens*, a devastating pest that feeds only on the phloem sap of rice plants. Molecular characteristic analysis indicated large variations in protein features and amino acid sequences among the predicted AAAP family members in BPH. Phylogenetic analysis clustered these AAAP transporters into three subgroups, with the members in the same group sharing a similar pattern of conserved motif distribution. Through ortholog gene recognition and spatiotemporal gene expression analysis, the AAAP gene *NlAAAP07*, which was predicted to regulate BPH larval growth and female fecundity, was identified. RNA interference (RNAi)-mediated suppression of *NlAAAP07* significantly postponed the duration of 3rd instar nymphs developing into adults from 7.4 days to 9.0 days, and decreased the oviposition amount and egg hatching rate of females by 30.7% and 11.0%, respectively. Our results provide a foundation for further functional analysis of AAAP transporters in BPH.

## 1. Introduction

Nitrogen is critical to an organism’s nutrition and energy metabolism, although its sources vary in different kingdoms of life [1,2]. Amino acids are the primary nitrogen sources for insect growth and development [1,3]. In addition to providing the raw materials for protein synthesis, amino acids have great physiological and biochemical importance in many other biological processes, including energy production, cell growth, nerve transmission, hormone secretion, and osmotic conditioning in insects [4,5,6]. However, most metazoans, including insects, lose their ability for de novo synthesis of the carbon skeletons of many essential amino acids during evolution and must acquire these amino acids through a dietary supply [7]. Among these, the free amino acids present in plant phloem serve as the sole supplier for phloem-feeding insects, irrespective of endosymbionts [3,8].

The uptake, transport, and distribution of dietary amino acids in insects rely on amino acid transporters (AATs) on the plasma membrane. With a few known exceptions, insect AATs can be classified into two major transporter families: the amino acid polyamine organocation (APC) family, and the amino acid auxin permease (AAAP) family [9,10]. As an ancient existing and functioning gene family, members of the APC family are ubiquitous in all life forms [11]. APC family proteins show significant variations in length from 350 to 800 aminoacyl residues and contain 12–14 transmembrane domains (TMDs) [12]. They catalyze the transportation of a wide spectrum of substrates into and out of living cells, including amino acids, polyamines, and metal ions [12,13]. In contrast, the AAAP family occurs exclusively in eukaryotes and possesses a relatively small number of aminoacyl residues, with most members having 11 putative TMDs [14,15]. Additionally, AAAP transporters have broader substrate specificity, accepting amino acids and conjugates, auxin (indole-3-acetic acid), and γ-aminobutyric acid [14]. Because of their distinct roles in neurotransmitter transport, the functional properties of the AAAP family members have been widely examined in recent years [16,17,18].

Since the first identification and cloning of γ-aminobutyric acid transporters in humans in 1990 [19], an increasing number of AAAP transporters has been recognized in a series of insect species, including *Drosophila melanogaster* [20,21], *Bombyx mori* [22], *Bemisia tabaci* [23], *Leptinotarsa* *decemlineata* [24], and several hemipterans belonging to Phylloxeridae [25]. Rapidly developing next-generation sequencing technology and bioinformatics algorithms are useful tools for identifying the AAAP transporters at a genome-wide level. For example, Xia, Yang, Gong, Xie, Pan, Guo, Zheng, Yang, Sun and Kang [23] presented a genome-wide investigation of the AAAP transporters in *B. tabaci* through homology searching and conserved functional domain recognition, yielding 25 putative AAAP transporters. Of these, two AAAP genes *BtAAAP15* and *BtAAAP21* were shown to be indispensable for *B. tabaci* survival by RNA interference (RNAi) assays.

In addition to catalyzing the cross-membrane flux of solutes, AAAP transporters can function in multiple life activities in insects, including virus resistance [26], host manipulation [25], and developmental regulation [27]. Among these, the roles of AAAP transporters as nutrient sensors to regulate insect growth, metamorphosis, and reproduction have been widely evaluated. For instance, the AAT of *pathetic* (*DmAAAP6*) mediates larval development, adult mass, and wing size by interfering with insulin/target of rapamycin (TOR) signaling cascades and modifying the titers of development-associated hormones in *D. melanogaster* [23,28]. Furthermore, considering the regulatory role of TOR pathways in egg development in insects, AAAP transporters may regulate insect fecundity [29]. However, compared with other insects, AAAP transporters from the brown planthopper (BPH), one of the most destructive insect pests of rice crops, are not well understood.

BPH, *Nilaparvata lugens* (Stål) (Hemiptera: Delphacidae), is a typical sap-sucking insect that feeds on rice phloem [30]. As a monophagous pest, BPH individuals nearly complete their life cycle in rice plants, causing severe damage to rice through direct sucking, ovipositing, and transmission of viruses [31,32]. With the help of endosymbionts, BPH can be well-adapted to unbalanced dietary amino acids, which have a significant effect on the growth and reproduction of BPH [33,34]. Thus, examining the mechanisms underlying amino acid transportation at the BPH/rice and BPH/endosymbiont interfaces is critical for managing this severe pest. However, genome-wide identification and functional characteristics of AAAP transporters in BPH have not been reported to date. 

In this study, we identified and characterized AAAP transporters based on the latest chromosome-wide genome assembly of *N. lugens* [35]. The conserved protein motifs, evolution characteristics, and spatiotemporal expression patterns of these putative AAAP members were analyzed. Moreover, the BPH AAAP transporter *NlAAAP07*, which potentially regulates larval growth and female reproduction in BPH, was identified and functionally verified using RNAi technology. Our results provide insights into the evolution and function of the AAAP transporter family in phloem-feeding insects, and suggest approaches for population management.

## 2. Materials and Methods

### 2.1. Insect Strains 

The insect strain of *N. lugens* used in this study was initially collected from a paddy field in Guangzhou (23.16° N, 113.27° E), Guangdong Province, China, in 2012. The laboratory population of BPH was maintained on rice seedlings of the Huanghuazhan variety in a climatically controlled chamber at a temperature of 26 ± 2 °C with a 16 h:8 h light/dark photoperiod and relative humidity of 80 ± 10%.

### 2.2. Search and Identification of AAAP Transporter Genes in BPH 

Putative AAAP family genes from BPH were obtained using two approaches: conserved protein domain identification, and basic local alignment search tool (BLAST) homology searching. For conserved domain recognition, a hidden Markov model (HMM) file of the AAAP gene family-specific domain of *Aa_trans* (accession number: PF01490) was downloaded from the Pram website (http://pfam.xfam.org/) (downloaded on 10 December 2020) and used to search against the BPH protein dataset, which was acquired from the insect genome database of InsectBase (http://www.insect-genome.com/) (downloaded on 10 December 2020). The HMM search was conducted using HMMER (v3.0), with an E-value cutoff of 10^−10^. A local BLASTP algorithm was also used to screen the potential AAAP gene family in the BPH genome with an E-value of 10^−5^. The AAAP family protein sequences from *D. melanogaster* and *Acyrthosiphon pisum* were used as queries. The protein sequences of AAAP genes were individually submitted to the SMART database (http://smart.embl.de/) to detect the presence of the characteristic domain of *Aa_trans* (Figure S1) (submitted on 15 December 2020). Finally, the coding sequences of these putative AAAP transporters in BPH were searched against the NCBI non-redundant protein (nr) database (https://www.ncbi.nlm.nih.gov/) to remove redundant sequences (searched on 20 December 2020). The resulting AAAP transporters were renamed depending on their location on BPH chromosomes. 

Using the same methods, AAAP transporter family members were also predicted in the genome of *Helicoverpa armigera*, which was acquired from the NCBI database (GenBank assembly accession: GCA_002156985.1). The resulting sequences of the AAAP transporter genes from cotton bollworm are listed in Table S1. 

### 2.3. Molecular Characteristics, Conserved Motif, and Chromosomal Assignment Analyses

To analyze the molecular characteristics of the putative AAAP proteins in BPH, we first predicted their respective molecular weights and isoelectric points using the ExPasy online tool (https://web.expasy.org/protparam/) (assessed on 5 February 2021). The number of TMDs in the putative AAAP proteins was determined using TMHMM (http://www.cbs.dtu.dk/services/TMHMM/) (analyzed on 6 February 2021). Additionally, we acquired the length and positional information of all 21 putative AAAP transporter genes from the GFF annotation file of the BPH genome. 

To predict the conserved motifs in BPH AAAP transporter proteins, we submitted the amino acid sequences to the MEME website (https://meme-suite.org/meme/) (analyzed on 7 February 2021), with the parameter of “the maximum number of motifs” set as 10. After the conserved protein motifs possessed by these AAAP transporters were deduced, TBtools software (v1.089) was employed to visualize the motif distribution pattern [36].

The chromosomal distribution of each putative AAAP gene in the BPH genome was determined using Mapchart (v2.32) software [37]. In addition, to analyze the gene syntenic and collinearity relationships among BPH AAAP family members, an MCScanX-based method was applied to detect two types of gene duplication events within this gene family, including tandem duplication and segmental duplication events [38].

### 2.4. Multiple Amino Acid Sequences Alignment and Phylogenetic Analysis of Putative AAAP Genes

The amino acid sequences of the *Aa_trans* domain possessed by each BPH AAAP protein were first identified using the Seqkit software (v0.12.0) [39], based on the resulting file of SMART analysis above. Multiple sequence alignments were subsequently conducted using Clustal W (v2.1) with the default parameters [40]. Finally, we used the software of Jalview (v2.11) to visualize final alignment results [41]. 

For phylogenetic analyses of the 21putative BPH AAAP family members, global alignment of the full-length amino acid sequences of these genes was initially carried out using the program Muscle (v3.8.31) [42]. The resulting file was utilized to construct a maximum likelihood (ML) phylogenetic tree using IQ-Tree (v2.0) [43], with the best-fit model of LG+F+R4 and ultrafast bootstrap of 1000 replicates. The acquired ML tree was further annotated using the EvolView (v3.0) online program (https://www.evolgenius.info/evolview/#login) [44] (assessed on 8 February 2021). 

Using the same procedure described above, we investigated the evolutionary relationship among the AAAP members from a selection of six insect species belonging to three orders, including Hemiptera (*N. lugens* and *B. tabaci*), Lepidoptera (*B. mori* and *H. armigera*), and Diptera (*D. melanogaster* and *Aedes aegypti*). The full-length protein sequences of AAAP transporters from *N. lugens* and *H. armigera* were predicted in this study. The protein sequences from the remaining four insects were downloaded from the NCBI database (https://www.ncbi.nlm.nih.gov/) (downloaded on 10 February 2021). To construct an unrooted ML evolutionary tree using IQ-Tree software, the best-fit model of LG+R7 was employed.

### 2.5. Developmental Stage- and Tissue-Specific Expression Profile Analyses of AAAP Genes

To reveal the temporal and spatial expression patterns of each identified BPH AAAP gene, we downloaded the transcriptome sequencing data of 21 groups of BPH from the NCBI SRA database (https://www.ncbi.nlm.nih.gov/sra) (assessed on 15 February, 2021), including eggs at 24 (E—24 h), 48 (E—48 h), and 120 h (E—120 h), first-instar nymph at 24 (1st—24 h) and 48 h (1st—48 h) after eclosion, second-instar nymphs at 24 (2nd—24 h) and 48 h (2nd—48 h) after ecdysis, third-instar nymphs at 24 (3rd—24 h) and 48 h (3rd—48 h) after ecdysis, fourth-instar nymphs at 24 (4th—24 h) and 48 h (4th—48 h) after ecdysis, fifth-instar nymphs at 24 (5th—24 h) and 48 h (5th—48 h) after ecdysis, and females at 24 (F—24 h) and 72 h (F—72 h) after eclosion, as well as six different tissues (head, salivary glands, integument, gut, fat body, and ovaries) from adults at 3 days after eclosion [45]. Kallisto (v0.46.1) software was then used to estimate the transcriptional levels of these putative AAAP genes in examined stages or tissues using the indicator of transcripts per million (TPM) values (Tables S2 and S3). The TPM values of each AAAP gene in the different stages and tissues were normalized by log2-transformations and displayed in two diagrams of the heatmap using the software TBtools software.

To confirm the validity of the spatiotemporal expressing expression pattern revealed by RNA-seq data, a total of 13 identified AAAP genes were selected for qPCR analysis in BPHs, which included 6 AAAP transporters in 8 different developmental stages (eggs at 48 h, first-instar nymphs at 24 h after eclosion, second-instar nymphs at 24 h after ecdysis, third-instar nymphs at 24 h after ecdysis, fourth-instar nymphs at 24 h after ecdysis, fifth-instar nymphs at 24 h after ecdysis, and female-adults at 72 h after eclosion), and 7 AAAP genes in 6 different tissues (gut, salivary gland, fat body, integument, ovary, and head) dissected from 72 h-female adults with the striatellus of shorted-winged and the body coloration of brown. For qPCR experiments, three independent biological replicates were performed for each developmental stage or tissue, and three technical replicates were conducted for each sample (see below).

### 2.6. Double-Stranded Rna (Dsrna) Synthesis and Injection to Bphs

For dsRNA synthesis, the DNA template for BPH was initially amplified by PCR using specific primers containing the T7 promoter sequence at both ends. The T7 RiboMAX Express RNAi System (Promega, Madison, WI, USA) and ds*NlAAAP07* (318 bp, GenBank accession number: XP_039285974) were synthesized from the purified DNA templates. We selected the dsRNA of green fluorescent protein (GFP) as a control. Subsequently, ds*NlAAAP07* and ds*GFP* were quantified using a NanoDrop 2000 instrument (Thermo Fisher Scientific, Waltham, MA, USA). A 1% agarose gel was used to examine the integrity of the synthesized dsRNA, which is shown in Table S4. 

dsRNA was administrated as previously described by Chen, et al. [46]. In the nymphal development observation experiments, the BPH nymphs of 3rd instar with similar size were collected from the culture chamber and anesthetized with CO_2_ for 15 s. Then, 60 nL of 2500 ng/μL ds*NlAAAP07* solution were injected into the mesothorax of each BPH placed on the icebox, with the microinjection of *dsGFP* as controls. Fifty nymphs were injected for each replicate, and three independent replicates were performed for each treatment. These injected insects were transferred to rice plants, and live samples were used to determine the RNA interference efficiency of ds*NlAAAP07* at 24, 48, and 72 h after dsRNA injection. With the same method, 1-day-old BPH females were selected to perform dsRNA injection in fecundity evaluation experiments. A total of 20 females was treated for each replicate, and three independent replicates were conducted per treatment. 

### 2.7. Total Rna Extraction and First-Strand Cdna Synthesis 

RNA isolation from collected BPH individuals (*n* ≥ 10) or dissected tissue samples (*n* ≥ 50) was performed using a Total RNA Kit II (Omega Bio-Tek, Norcross, GA, USA) following the manufacturer’s protocol. Each extracted RNA sample was quantified using the RNA 6000 Nano LabChip kit and Bioanalyzer 2100 (Agilent Technologies, Santa Clara, CA, USA), and sample integrity was determined by 1.2% agarose gel electrophoresis. First-strand cDNA was synthesized from 1 μg of total RNA using a PrimeScript™ RT reagent kit (Takara Bio, Inc., Otsu, Japan), according to the manufacturer’s instructions. Three dsRNA-injected samples were randomly selected to perform RNA extraction for each replicate, and three independent replicates were conducted per treatment.

### 2.8. Quantitative Real-Time PCR (qPCR) Analyses

The qPCR primer for examined AAAP genes was designed using Primer Premier (v5.0) software (Premier Biosoft, Rockville, MD, USA) (Table S4). To ensure the specific amplification of the intended target genes in BPHs, all qPCR primers used in the following study were firstly checked using the online software tool of Primer-BLAST (http://www.ncbi.nlm.nih.gov/tools/primer-blast) prior to qPCR experiments [47] (checked on 15 February 2021). The qPCR system of the 10 μL reaction sample consisted of 1 μL cDNA, 0.3 μL forward and reverse primers (concentration of 10 μmol/L), 5 μL SYBR FAST Universal qPCR mix (KAPA Biosystems, Woburn, MA, USA), and 3.4 μL ddH_2_O. qPCR assays were conducted using a Light Cycler 480 System (Roche Diagnostics, Basel, Switzerland) and the SYBR FAST Universal qPCR Kit (KAPA Biosystems, Woburn, MA, USA), with a PCR amplification procedure as follows: one cycle at 95 °C for 5 min, followed by 45 cycles at 95 °C for 10 s, 60 °C for 20 s, and 72 °C for 20 s. For the entire experiment, three biological replicates and three technical replicates were used for each treatment. 

### 2.9. Bioassays

Live BPH nymphs at 3rd instar injected with ds*NlAAAP07* or ds*GFP* were individually maintained on a three-leaf-stage rice seedling of the Huanghuazhan variety growing in a transparent polycarbonate jar (diameter 20 cm, height 90 cm). The survival status and developmental ages of the BPHs were recorded daily until the adult stage. Thereafter, 1-day-old BPH females from both groups were individually weighed using a microbalance. Twenty independent replicates were performed for each treatment throughout the experiment. 

Based on our previous results [46,48], we measured the fecundity of dsRNA-injected BPHs within the first 7 days to evaluate the effect on BPH fertility of dsRNA administration. Briefly, awakened female adults treated with dsRNA were paired with two untreated males and transferred to the rice plants described above. All adults were removed from the rice seedlings 7 days later. The number of newly hatched nymphs was recorded, and the nymphs were removed every day until no nymphs were observed for 4 consecutive days. Next, we dissected the plants to count the unhatched eggs. The sum of the nymphs and unhatched eggs was used as the total egg-laying count for each female. Based on the values of nymph number and egg amount, egg hatchability was determined. Twenty independent trials were conducted for the ds*GFP*- and ds*NlAAAP07*-injected groups. 

### 2.10. Statistical Analyses

For the statistical analysis of the qPCR results, all mRNA levels were normalized to that of *β-actin* in the corresponding samples. The method of 2^−^^△△Ct^ was used to evaluate the relative transcriptional levels of AAAP genes for different treatment. The statistical difference in mRNA levels of examined genes among multiple developmental stages and tissues were determined by one-way ANOVA followed by Duncan’s multiple range test using SPSS (v17.0) software (SPSS Inc., Chicago, IL, USA), with a *p*-value-cut off of 0.05. Student’s *t*-test was used to determine the significance of difference in the transcriptional levels of *NlAAAP07*, growth and development indices of nymphs (including the survival rates, development duration, and body weights of newly emerged adults), and fecundity indicators (including egg number, nymph number, and egg hatching rate) between the ds*NlAAAP07*-treated and ds*GFP*-treated groups. *p* < 0.05, indicates statistical significance and is marked by *, whereas *p* < 0.01 was considered to be highly significant and is marked by **. All analyses were performed using the SPSS software (v17.0), and the results are presented as the mean ± standard error (SE).

## 3. Results

### 3.1. Identification and Molecular Characteristics of AAAP Transporter Gene in BPH

**Figure 1:**
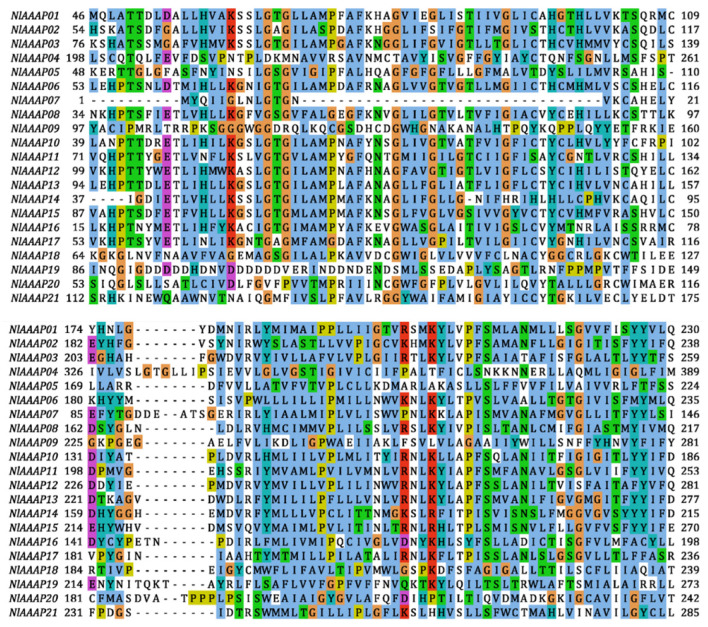
Amino acid sequence alignments of *Aa_trans* domains from the 21 identified AAAP transporters in BPH. The amino acid sequences encoding the *Aa_trans* domains of putative AAAP transporters were extracted from the BPH protein dataset and aligned using Clustal W (v2.1). Jalview (v2.11) was used to visualize the final alignment results.

Through a combined homology searching and conserved functional domain recognition, AAAP transporters were screened in the BPH genome. A set of 21 candidate AAAP transporter genes was identified (Table S1 and Table 1) and named *NlAAAP01*-*NlAAAP21,* based on its position in the BPH genome (Table 1). All identified BPH AAAP proteins were found to possess the *Aa_trans* (PFAM database accession: PF01490) AAAP family featuring domain (Figure S1). Molecular characteristics analysis showed that these verified AAAP proteins were encoded by 416–928 amino acid residues, with a deduced molecular weight ranging from 45.70 to 102.66 kDa and a theoretical isoelectric point (pI) ranging from 4.98 to 9.31 (Table 1). In addition, protein secondary structure analysis revealed that the number of TMDs in these putative AAAP transporter proteins was between 7 and 21, with 9–12 being the most abundant (Table 1). Furthermore, from the alignment of full-length amino acid sequences, these putative AAAP proteins showed high variability in the amino acid identity of the *Aa_trans*-type domains, with few conserved amino acid residues observed among most AAAP genes in BPH (Figure 1).

### 3.2. Evolutionary Relationship and Conserved Motif Analysis of AAAP Transporters in BPH

**Figure 2:**
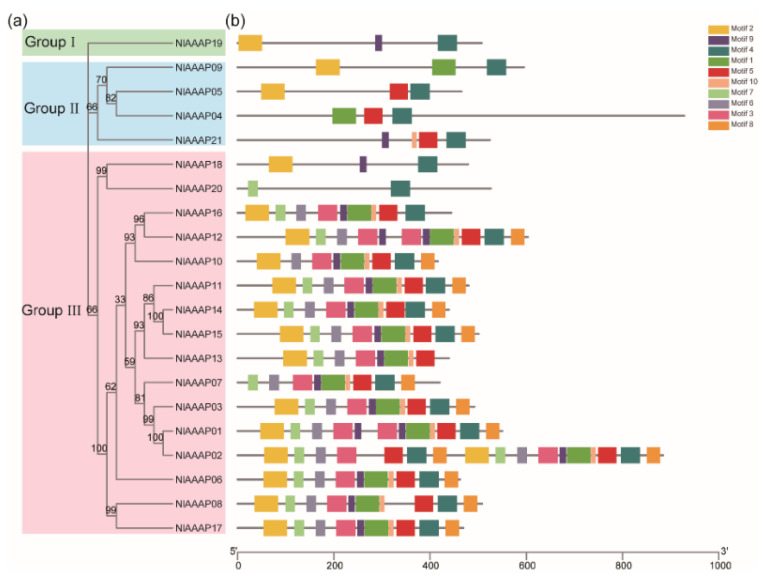
Phylogenetic analysis and conserved motif prediction of putative AAAP transporter genes in BPH. (**a**) Phylogenetic tree of the 21 identified AAAP transporter genes from BPH. The maximum likelihood (ML) evolutionary tree was built by IQ-Tree (v2.0) based on the best-fit model of LG+F+R4, and the number at nodes indicated the bootstrap value from 1000 iterations. (**b**) Motif composition of AAAP transporters in BPH. MEME Suite (v5.5.3) was employed to predict the conserved motifs possessed by each BPH AAAP transporter. Ten motifs were finally identified and are represented by boxes with different colors.

To explore the phylogenetic relationship among predicted AAAP transporters in BPH, an ML tree was produced by IQ-Tree software using the full-length amino acid sequence of these members (Figure 2a). During ML tree construction, the best-fit model of LG+F+R4 and 1000 bootstrap tests were applied. Based on phylogenetic analysis, these putative AAAP transporters were grouped into three clades, groups I, II, and III), containing 1, 4, and 16 members, respectively (Figure 2a). 

Thereafter, the distribution pattern of conserved motifs in these AAAP genes was analyzed using the MEME suite. MEME analysis identified 10 conserved motifs in 21 putative BPH AAAP genes, which were designated as motifs 1–10 (Figure 2b). Some motifs were widespread in most AAAP transporters, particularly motif 4, which are present in 20 AAAP transporters of BPH. Therefore, it was assumed to be a characteristic motif of the AAAP gene family. In addition, although the number and types of conserved motifs varied significantly among AAAP transporters, some genes on the same branches in our ML tree showed a similar composition in conserved motifs. For example, compared with groups I and II, each member in group III, except for *NlAAAP18* and *NlAAAP20*, contained a larger number of conserved motifs (Figure 2b). 

### 3.3. Chromosomal Location and Synteny Analysis of AAAP Gene Family Members in BPH

**Figure 3:**
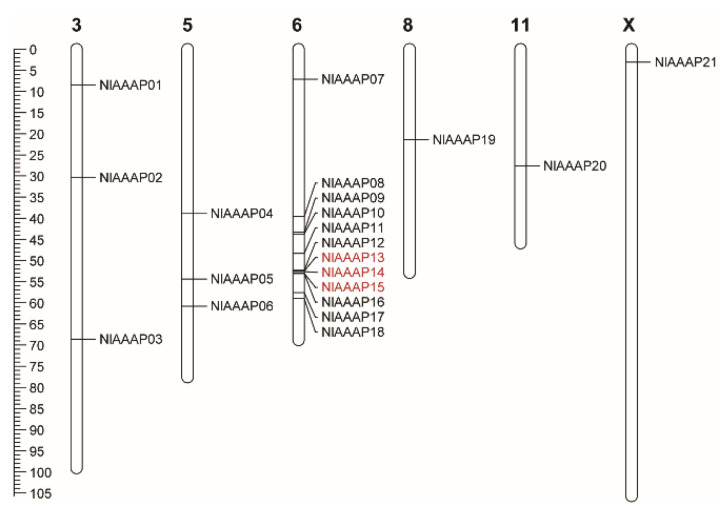
Chromosomal distribution and synteny analysis of AAAP transporter genes in the BPH genome. All the identified AAAP genes were mapped onto the BPH chromosomes using TBtools software based on the genome annotation document. Gene duplication analyses were performed using MCScanX software. Two gene pairs of tandem duplication were identified within BPH AAAP family, which are marked by a red box. No segmental gene duplication was found for putative AAAP transporters. The length of each chromosome is indicated by the sale on the left.

We subsequently positioned the AAAP transporters onto their respective chromosomes and analyzed potential gene duplication events during their evolutionary process. The results suggested that 21 putative AAAP genes were unevenly distributed on six out of 16 chromosomes of the BPH genome, including 5 autosomes (Chr 3, 5, 6, 8, and 11) and 1 sex chromosome (Chr X), with the number on each chromosome varying from 1 to 12 (Table 1; Figure 3). More than half of the putative BPH AAAP transporter genes (12 of 21) were concentrated within the region of 20 MB on Chr 6, indicating a potential gene duplication event during AAAP gene expansion in BPH. Through collinearity analysis using MCSanX software, two pairs of putatively tandem duplicated genes were detected among our predicted BPH AAAP transporters, including *NlAAAP13*-*NlAAAP14* and *NlAAAP14*-*NlAAAP15* (Figure 3). In contrast, no segmental duplication pairs of AAAP genes were identified in the BPH genome.

### 3.4. Phylogenetic Analysis of AAAP Family Members from Six Insect Species in Different Orders

**Figure 4:**
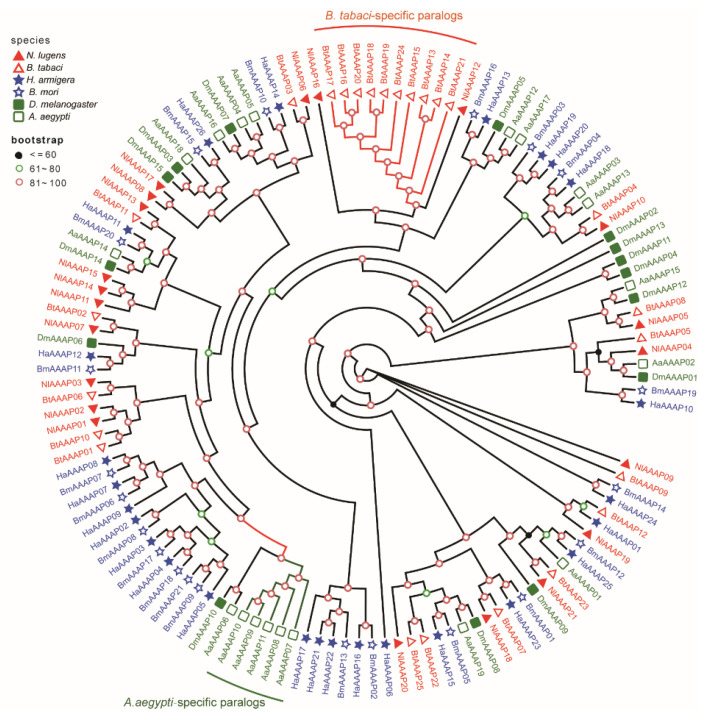
Phylogenetic relationship among AAAP gene family members from six different insect species belonging to three orders. The predicted full-length protein sequences of AAAP transporters in *N. lugens*, *B. tabaci*, *B. mori*, *H. armigera*, *D. melanogaster*, and *A. aegypti* were acquired from their respective protein datasets. The phylogenetic tree comprising 127 AAAP family members was constructed using IQ-Tree software (v2.0) with the maximum likelihood (ML) method with 1000 bootstrap replicates. Genes from the same insect order (Hemiptera, Lepidoptera, and Diptera) are represented by the same colors.

To reveal the evolutionary relationship among AAAP family members in insects, multiple amino acid sequence alignments were performed on one hundred and twenty-seven putative AAAP transporters from six representative species of three insect orders (Hemiptera: *N. lugens* and *B. tabaci*; Lepidoptera: *B. mori* and *H. armigera*; Diptera: *D. melanogaster* and *A. aegypti*). The resulting alignment file was utilized to construct an unrooted ML phylogenetic tree based on the best-fit model of LG+R7. Most AAAP transporters from the same insect order were clustered into one clade (Figure 4), which was consistent with the taxonomic classification of insects. Moreover, some insects exhibited species-specific expansion of AAAP family genes. For example, there were five and ten paralogs for *A. aegypti-* and *B. tabaci*-specific expansion of AAAP transporters, respectively (Figure 4). 

### 3.5. Spatiotemporal Expression Profiling of AAAP Genes in BPH

**Figure 5:**
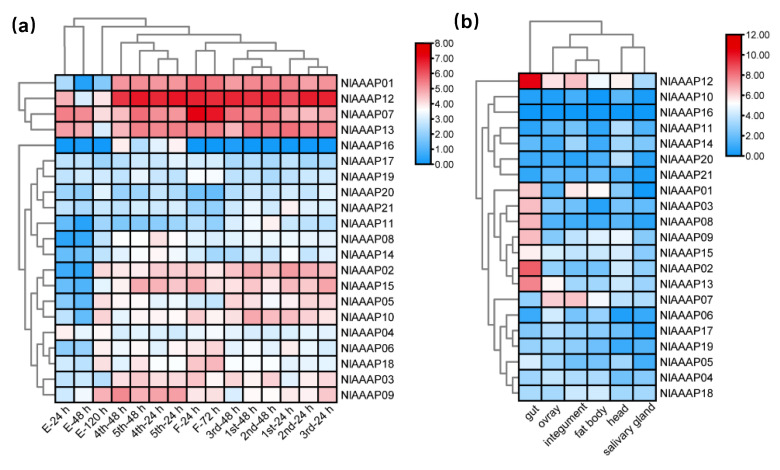
Spatiotemporal expression pattern of putative AAAP transporter genes in BPH. (**a**) Heatmap of developmental stage-specific expression of BPH AAAP genes in the egg, nymphal, and adult stages. (**b**) Heatmap of tissue-specific expression profiling of AAAP transporters in the head, salivary glands, integument, gut, fat body, and ovary of BPH. RNA-seq data of the 21 different stages or tissues in BPH was downloaded from the NCBI database, and the TPM (transcript abundance in transcripts per million) value for each AAAP gene was calculated using Kallisto (v0.46.1). All data were log2 transformed before the heatmap diagrams were generated using TBtools software. High and low levels of gene expression are represented by red and blue, respectively.

To investigate the age- and tissue-specific expression profiles of AAAP genes in BPH, large-scale RNA-seq data at fifteen different developmental stages (three stages in the egg period, ten in the nymph period, and two in the adult period) and in six different tissues (head, salivary glands, integument, gut, fat body, and ovary) in BPH were downloaded from the NCBI database and analyzed using Kallisto (v0.46.1) software. Using the stage-specific expression analysis results, all 21 identified BPH AAAP genes were classified into two groups based on their global expression patterns at different developmental stages (Figure 5a), with *NlAAAP01*, *NlAAAP07*, *NlAAAP12*, and *NlAAAP13* showed significantly higher expression levels at most stages than those of the remaining 17 AAAP genes. Additionally, most BPH AAAP genes had relatively lower transcript abundance in the egg period stage than in the nymph and adult stages. 

Similarly, BPH AAAP transporters showed divergent expression patterns in different tissues in BPH (Figure 5b). For instance, 42.9% (9 of 21) of AAAP genes reached their highest expression levels in the gut tissue, whereas *NlAAAP16* was mainly expressed in the tissues of the fat body and ovary and was not detected in the other three examined tissues. Furthermore, we counted the number of genes whose expression levels peaked in tested tissues. The results showed that gut tissue possessed the highest number of highly expressed genes (9), followed by the ovary (4), head (4), fat body (3), and integument tissue (1). However, no AAAP transporters showed the highest transcriptional levels in the salivary glands. 

In addition, to verify the transcriptional expression profiles of AAAP genes indicated by RNA-seq data, a total of 13 AAAP transporter genes were selected to perform qPCR analysis, including 7 genes in different developmental stages (eggs, 1st instar nymphs, 2nd instar nymphs, 3rd instar nymphs, 4th instar nymphs, 5th instar nymphs, 24 h-female adults, and 72 h-female adults) and 6 genes in different tissues (head, salivary glands, integument, gut, fat body, and ovary) dissected from 72 h-female adults. The results showed concordant expression patterns of BPH AAAP genes between RNA-seq data and qPCR analysis (Figures S2 and S3).

### 3.6. Effect of Silencing NlAAAP07 on the Growth of BPH Nymphs

The AAAP gene of *DmAAAP06* plays an important role in modulating the developmental process of *D. melanogaster*. Therefore, we surveyed the orthologous genes of *DmAAAP06* in five other insect species. In our phylogenetic tree, *BtAAAP02*, *NlAAAP07*, *HaAAAP12*, and *BmAAAP11* were clustered together with *DmAAAP06* (Figure 4), indicating their role in the growth regulation of their respective insects. Accordingly, the function of *NlAAAP07* in regulating BPH growth was verified using RNAi technology. 

**Figure 6:**
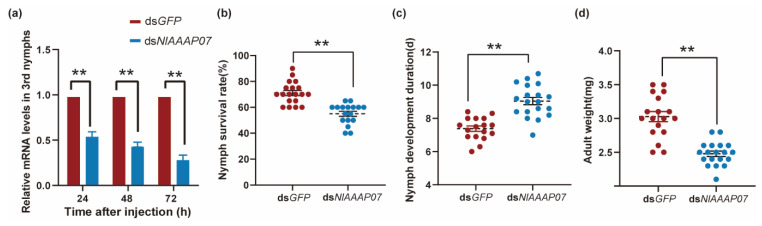
Effect on nymphal growth of RNAi knockdown of *NlAAAP07* in BPH. (**a**) Effect of ds*GFP* and ds*NlAAAP07* administration on relative expression levels of *NlAAAP07* in 3rd instar nymphs at 24, 48, and 72 h post-RNAi; The influence of ds*NlAAAP07* administration on the survival rate (**b**) and developmental duration (**c**) of BPH nymphs from 3rd instar growing into adults; (**d**) Influence of ds*NlAAAP07* treatment on the body weights of newly emerged adults. Student’s *t*-test was used to assess the significance of the difference in nymphal growth indicators of BPH between ds*GFP*- and ds*NlAAAP07*-treated groups (**, *p* < 0.01). Values are the mean ± SE from 20 independent biological replicates.

The interference efficiency by ds*NlAAAP07* was determined using qPCR assays, which showed that ds*NlAAAP07* treatment significantly reduced the transcript levels of *NlAAAP07* in BPH nymphs by 44.0%, 55.0%, and 70.0% at 24, 48, and 72 h post-treatment, respectively, relative to those in the control group (injected with ds*GFP*) (all *p* < 0.05, *t*-test; Figure 6a). Furthermore, administration of ds*NlAAAP07* significantly reduced the survival rates of BPHs from third-instar nymphs to adults, with 71% in the ds*GFP*-treated group vs. 55% in the ds*NlAAAP07*-treated group (*p* = 0.001, *t*-test; Figure 6b), and markedly postponed their developmental duration from 7.4 to 9.0 days (*p* = 0.001, *t*-test; Figure 6c). Moreover, compared with the control group, the body weight of newly emerged adults was significantly downregulated by ds*NlAAAP07* treatment (*p* < 0.001, *t*-test; Figure 6d). These results indicate that *NlAAAP07* might be involved in the developmental regulation in BPH nymphs.

### 3.7. Effect of Silencing NlAAAP07 on the Reproductive Regulation in BPH Females

In addition to high expression in the integument and fat body tissue, the BPH AAAP gene of *NlAAAP07* also showed abundant transcript levels in the adult stage and ovary tissue, as revealed by RNA-seq analysis (Figure 5a,b). Therefore, this gene may also regulate the reproduction of BPH, and a verification experiment based on RNAi was conducted. 

**Figure 7:**
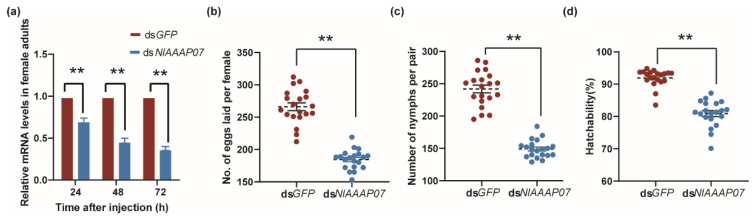
Effect on the BPH fecundity of RNAi knockdown of *NlAAAP07*. (**a**) Effect of ds*GFP* and ds*NlAAAP07* administration on relative expression levels of *NlAAAP07* in females at 24, 48, and 72 h post-RNAi; The influence of silencing *NlAAAP07* on the egg-laying amount (**b**), nymph number (**c**), and egg hatching rate (**d**) in BPH. The significance of the difference in BPH fecundity indexes between treatments was determined by Student’s *t*-test (**, *p* < 0.01), and the data are represented as the mean ± SE (*n* = 20).

Administration of ds*NlAAAP07* resulted in a significant reduction in *NlAAAP07* expression in BPH adults at 24 h (29%), 48 h (53%), and 72 h (62%) after microinjection of dsRNA, when compared with that in the control BPHs treated with ds*GFP* (Figure 7a). In addition, silencing of *NlAAAP07* significantly downregulated the fecundity of BPH. The numbers of eggs laid, number of nymphs, and hatching rate of BPH eggs showed marked decrease of 30.7%, 37.1%, and 11.0%, respectively (all *p* < 0.001, *t*-test; Figure 7b–d), compared with those in control group. These findings suggest that the amino acid transporter gene *NlAAAP07* regulates BPH fecundity.

## 4. Discussion

AAAP transporters play a crucial role in a wide range of physiological processes, including solute transport, osmotic conditioning, neuronal nutrition, cellular growth, and differentiation [27,28,49,50]. However, their molecular features and biological functions remain unclear in most phloem-feeding insects [32]. In this study, a genome-wide analysis of the AAAP gene family was performed in BPH, a disastrous pest for rice crops. Twenty-one putative AAAP transporters were identified in the BPH genome, and their protein characteristics and evolutionary patterns were comprehensively analyzed. A candidate AAAP transporter gene, *NlAAAP07*, was further identified by molecular phylogenetic analysis and gene expression profiling and predicted to regulate BPH growth and reproduction. The biological function of *NlAAAP07* was validated by RNAi and bioassay experiments. 

### 4.1. Molecular Characteristics and Evolutionary Pattern of AAAP Transporters in BPH 

In this study, 21 putative AAAP transporters were identified in BPH (Table 1; Figure S1). This value is lower than that in *Manduca sexta* (26, Lepidoptera), *B. tabaci* (25, Hemiptera), and *A. pisum* (23, Hemiptera), equal to those in *B. mori* (21, Lepidoptera), and greater than those in *A.s aegypti* (19, Diptera), *Apis mellifera* (16, Hymenoptera), *D. melanogaster* (15, Diptera), and *Nasonia vitripennis* (14, Hymenoptera) [23], indicating significant variation in the size of the AAAP family among different insect species. Based on the finding of previous reports [23,51], we preliminarily predicted that lepidopteran and hemipteran insects had more AAAP transporters than those in dipterans and hymenopterans. However, further evidence is needed to support this hypothesis. 

Although a characteristic domain of *Aa_trans* was predicted to be present in all putative AAAP transporter proteins in BPH, relatively low sequence conservation was found for this domain from different AAAP transporters (Figure 1). The limited sequence similarity among AAAP family proteins was also reported by Young, Jack, Smith and Saier Jr [14] and Jack, Paulsen and Saier [15]. A distantly related pattern among AAAP family members, resulting from a long divergence history, may be responsible for their varied substrate specificity [14]. In contrast, conserved protein motif analysis demonstrated that AAAP members who fell into the same cluster in our ML phylogenetic tree had a similar motif distribution pattern (Figure 2a,b), suggesting that these AAAP transporters share specific functional properties.

Gene duplication is considered one of the key evolutionary drivers of genes and genomes, providing raw genetic materials for the origin of physiological or morphological novelties [52,53]. Three types of gene duplication events can occur during organismal evolution: segmental duplication, tandem duplication, and transposon-mediated retroposition [54]. Tandem duplication is more common in the gene family expansion of diploid animals than in that of plants, resulting in a series of homologous genes closely arranged in the same or adjacent genomic regions [52]. Our gene duplication analysis identified two tandem duplication gene pairs within the AAAP family in BPH (Figure 3). Tandem duplication of AAAP transporters has also been reported in many other animals [23,55,56]. However, the amino acid identity of paralogous sequences among gene copies was lower in BPH than in other animals, as revealed by this study (Figure 1), suggesting an ancient divergence of AAAP members by tandem duplication during BPH evolution.

### 4.2. Spatiotemporal Expression Pattern of AAAP Transporter in BPH

Determining the spatiotemporal expression pattern is vital for elucidating the functional roles of genes [54]. In this study, the transcriptional expression of putative AAAP family members was profiled at 15 different life stages and in six different BPH tissues (Figure 5a,b). In stage-specific gene expression analysis, most BPH AAAP transporters exhibited significantly lower transcriptional levels in the egg stage than in the nymphal and adult stages (Figure 5a). This result is consistent with that of a previous report on *B. tabaci* [23]. Considering amino acid uptake and transport events occur mainly in the stages of nymphs and adults, the two feeding stages of BPH, it was reasonable that few AAAP transporters showed high levels of expression in the egg stage. In addition, the expression levels of four AAAP transporters (*NlAAAP01*, *NlAAAP07*, *NlAAAP12*, and *NlAAAP13*) were significantly higher in the nymphal and adult stages than in the other stages, indicating some vital roles in the fundamental life processes of BPH [23]. 

Tissue-specific expression pattern analysis demonstrated that the gut possessed the maximum number of AAAP transporters, reaching their highest transcriptional levels in the corresponding tissue (9), followed by the head (4), ovary (4), fat body (3), and integument (1) (Figure 5b). This result indicates that the tissues mentioned above are the main functional sites of BPH AAAP transporters. First, as the largest part of the digestive tract in insects, the midgut is generally considered the major region of food digestion and nutrient absorption [57]. Therefore, it was expected that most AAAP transporters were expressed at high levels in the gut tissue, which has been widely observed in many other insect species and nematodes [24,58,59]. In addition, AAAP transporters showing a high level of expression in the head of BPHs, containing the central nervous system of the brain, may mediate the biosynthesis and transportation of monoamine neurotransmitters [59,60]. Moreover, the fat body is an amino acid-sensitive organ that coordinates insect growth and development [20], and the ovary is the most important reproductive organ in insects. Accordingly, AAAP transporters displaying high expression in the BPH fat body and ovary may be involved in regulating BPH growth and reproduction. 

### 4.3. Involvement of NlAAAP07 in Regulating BPH Growth and Reproduction

Apart from solute transport, AATs can control insect growth and reproduction by sensing amino acid availability in extracellular media [20,55]. Silencing the expression of an AAAP transporter of *slimfast* (*slif*) led to global growth restriction in *D. melanogaster*, similar to that observed in case of amino acid deficiency [20]. RNAi-mediated knockdown of cationic amino acid transporter genes significantly reduced the oviposition amount of *A. aegypti* and vitellogenin gene expression [61,62]. In this study, we identified the BPH AAAP gene *NlAAAP07*, which has dual functions in modulating BPH growth and fecundity.

Based on our molecular phylogenetic analysis, *NlAAAP07* was identified as the orthologous gene of *DmAAAP6* (CG3424) (Figure 3), which is a potent mediator of growth in *D. melanogaster* [62]. Silencing of *NlAAAP07* expression resulted in a significantly prolonged developmental duration and increased mortality of 3rd instar nymphs growing into adults, accompanied by a significantly reduced body weight of females (Figure 6b–d). Previous studies revealed that AAAP transporters can operate as nutrient sensors that respond to local amino acid levels in multicellular organisms. By interacting with IR/TOR signaling components, these AAAP transporters can modify the titers of endocrine hormones that control insect growth and reproduction [20,28]. For instance, Fu, Guo, Ahmat and Li [24] reported that RNA interference against the AAT gene of *LdNAT1* retarded larval growth and impaired pupation in *L. decemlineata*, significantly repressing the IR/TOR signaling pathway and decreasing 20-hydroxyecdysone and juvenile hormone levels. However, further studies are needed to confirm whether *NlAAAP07* controls larval development via the IR/TOR pathways and hormonal mechanisms in BPH.

RNAi knockdown of *NlAAAP07* significantly decreased the number of eggs deposited, nymph counts, and egg hatchability of BPH (Figure 7b–d), suggesting the crucial role of *NlAAAP07* in regulating of BPH fecundity. Although there is little direct evidence for the involvement of AAAP transporters in the regulation of BPH fecundity, the regulatory effect of APC transporters on mosquito fertility via amino acid-induced TOR signaling has been widely explored in *A. aegypti* [61,62,63]. We hypothesized that the AAAP transporter *NlAAAP07* mediates BPH reproduction through a similar mechanism, which must be confirmed in further studies. 

In addition, it is worth noting that besides *NlAAAP07*, *NlAAAP12* and *NlAAAP13* also reached their high levels of expression in the ovary of 3-day-old female adults, indicating some potential roles of the two gene in regulating BPH fecundity (Figure 5b). Furthermore, *NlAAAP12* and *NlAAAP13* highly expressed in the gut, which may be directly related to amino acid ingestion from diets and serve as nutritional sensors to affect vitellogenesis and egg maturation via the insulin/TOR signaling cascades [61,64]. Therefore, a deep exploration is essential on the functions of the two AAAP transporters in the reproductive regulation in BPH. 

## 5. Conclusions

Genome-wide identification and characterization of AAAP family members were performed on the BPH genome, yielding 21 putative AAAP transporter genes that were nonrandomly distributed on six chromosomes. Phylogenetic and conserved motif analysis showed that although there was a large variation in the protein sequences of specific *Aa_trans* domains from different AAAP transporters, the BPH AAAP family members clustered into the same subgroup by the evolutionary tree share a high similarity in motif distribution patterns. Tandem duplication events contributed to the expansion the AAAP family in BPH, based on gene duplication analysis. In addition, through homology analysis of the evolutionary pattern and spatiotemporal expression profiling, the AAAP gene *NlAAAP07,* presumed to modulate BPH larval growth and female fecundity, was identified and validated using RNAi and bioassay experiments. Our results will provide insights for the functional analysis of AAAP transporters in BPH and help in the development of new approaches for its integrated management.

## Figures and Tables

**Table 1:** Identification and molecular characteristics of AAAP transporters in the BPH genome.

Gene Name	Genome Identifier	Locus			CDS	TMDs	Mw (kDa)	pI	Strand
		Chr.	Starting	Ending					
*NlAAAP01*	*Nlug16008-TA*	Chr3	8548901	8568080	550	11	60.49	8.93	−
*NlAAAP02*	*Nlug07328-TA*	Chr3	30440085	30471421	884	21	96.76	9.31	−
*NlAAAP03*	*Nlug13074-TA*	Chr3	68618099	68628120	492	11	54.53	7.78	−
*NlAAAP04*	*Nlug04460-TA*	Chr5	38883176	38895525	928	9	102.66	5.81	+
*NlAAAP05*	*Nlug00688-TA*	Chr5	54447610	54457796	465	11	51.39	7.08	−
*NlAAAP06*	*Nlug19955-TA*	Chr5	60830132	60841982	462	9	50.63	8.30	−
*NlAAAP07*	*Nlug15359-TA*	Chr6	7100334	7114380	420	9	46.00	4.98	+
*NlAAAP08*	*Nlug12813-TA*	Chr6	39594747	39612213	508	10	56.59	8.51	+
*NlAAAP09*	*Nlug01999-TA*	Chr6	43302072	43322579	595	11	67.43	8.82	−
*NlAAAP10*	*Nlug07975-TA*	Chr6	43719019	43725694	416	10	45.70	8.34	−
*NlAAAP11*	*Nlug10573-TA*	Chr6	48272935	48278441	480	10	53.08	8.40	+
*NlAAAP12*	*Nlug01820-TA*	Chr6	52347198	52362195	603	12	66.90	7.18	−
*NlAAAP13*	*Nlug01818-TA*	Chr6	52476165	52496749	439	7	48.24	8.45	+
*NlAAAP14*	*Nlug01815-TA*	Chr6	52498817	52525317	439	7	48.58	9.19	−
*NlAAAP15*	*Nlug01817-TA*	Chr6	52525911	52547690	501	10	55.47	8.24	+
*NlAAAP16*	*Nlug01797-TA*	Chr6	53003412	53014473	444	10	50.01	6.65	+
*NlAAAP17*	*Nlug07143-TA*	Chr6	57602062	57651016	469	10	51.92	8.33	+
*NlAAAP18*	*Nlug10388-TA*	Chr6	59044977	59087443	479	11	52.81	6.81	−
*NlAAAP19*	*Nlug10180-TA*	Chr8	21356573	21375023	507	10	56.38	5.39	+
*NlAAAP20*	*Nlug02695-TA*	Chr11	27606288	27620896	526	7	58.36	8.60	−
*NlAAAP21*	*Nlug04947-TA*	ChrX	3124780	3137143	524	10	59.06	8.03	−

Chr., Chromosome; CDS, Coding sequence; TMDs, Transmembrane domains; MW, Molecular weight; pl, Isoelectric point; +, the sense strand; −, the antisense strand.

## Data Availability

The data presented in this study are available in Supplementary File.

## Supplementary Materials

The following are available online at https://www.mdpi.com/article/10.3390/insects12080746/s1. Figure S1: Domain organizations of putative AAAP transporters in BPH. Figure S2: Stage-specific expression patterns of some selected AAAP transporters. The mRNA levels of *NlAAAP01* (A), *NlAAAP02* (B), *NlAAAP07* (C), *NlAAAP09* (D), *NlAAAP12* (E), *NlAAAP13* (F), and *NlAAAP16* (G) in different developmental stages were determined by qPCR analysis. The mRNA level in the egg was set to 1. All mRNA levels were normalized relative to those of *β-actin* in the corresponding samples. The data are represented as the mean ± SE (*n* = 3). Different low-case letters above the columns indicated significant differences (*p* < 0.05, Duncan’s multiple range test). Figure S3: Tissue-specific expression profiles of some selected AAAP transporters. The mRNA levels of *NlAAAP01* (A), *NlAAAP06* (B), *NlAAAP07* (C), *NlAAAP16* (D), *NlAAAP18* (E), and *NlAAAP20* (F) in different tissues were determined by qPCR analysis. The mRNA level in the head was set to 1. All mRNA levels were normalized relative to those of *β-actin* in the corresponding samples. The data are represented as the mean ± SE (*n* = 3). Different low-case letters above the columns indicated significant differences (*p* < 0.05, Duncan’s multiple range test). Table S1: Predicted protein sequences of AAAP transporters identified in *Nilaparvata lugens* and *Helicoverpa armigera*. Table S2: TPM values of putative AAAP transporter genes in BPH at 15 different developmental stages. Table S3: TPM values of putative AAAP transporter genes in BPH from six different tissues. Table S4: Primers used for qPCR and RNAi experiments.
