# Supplementary material for: Genome-Wide Analysis of the Amino Acid Auxin Permease (AAAP) Gene Family and Identification of an AAAP Gene Associated with the Growth and Reproduction of the Brown Planthopper, Nilaparvata lugens (Stål)
Source: Insects. 2021 Aug 18;12(8):746. doi: 10.3390/insects12080746 (PMC8397168; doi:10.3390/insects12080746)
Supplement: Supplementary file 1 [file insects-12-00746-s001.zip › Supplementary Figures.pdf]

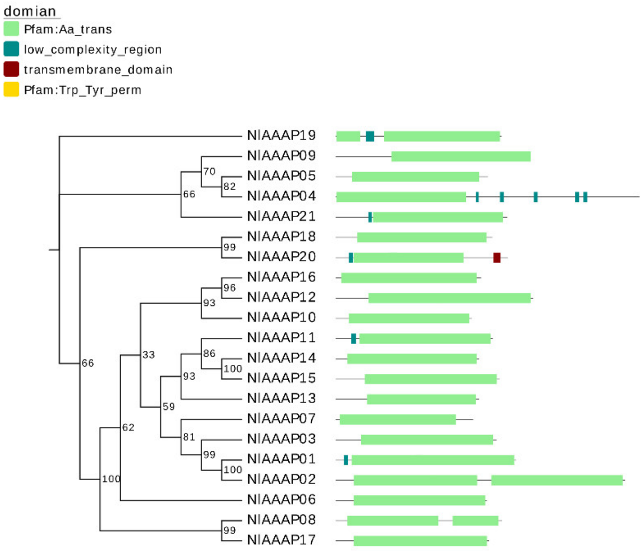

Figure S1: Domain organizations of putative AAAP transporters in BPH.

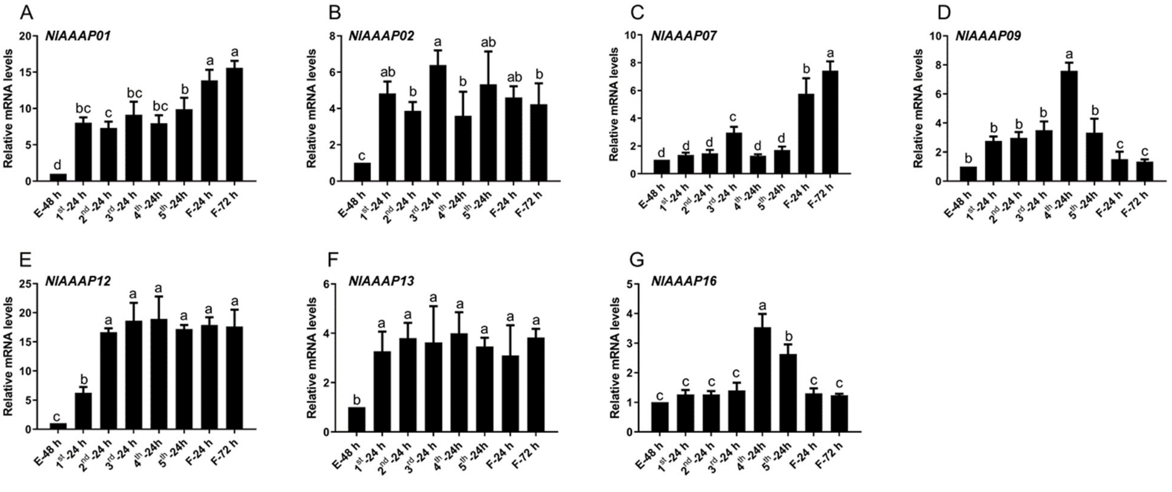

Figure S2: Stage-specific expression patterns of some selected AAAP transporters. The mRNA levels of *NlAAAP01* (A), *NlAAAP02* (B), *NlAAAP07* (C), *NlAAAP09* (D), *NlAAAP12* (E), *NlAAAP13* (F), and *NlAAAP16* (G) in different developmental stages were determined by qPCR analysis. The mRNA level in the egg was set to 1. All mRNA levels were normalized relative to those of *β-actin* in the corresponding samples. The data are represented as the mean ± SE (n = 3). Different low-case letters above the columns indicated significant differences ($P < 0.05$, Duncan's multiple range test).

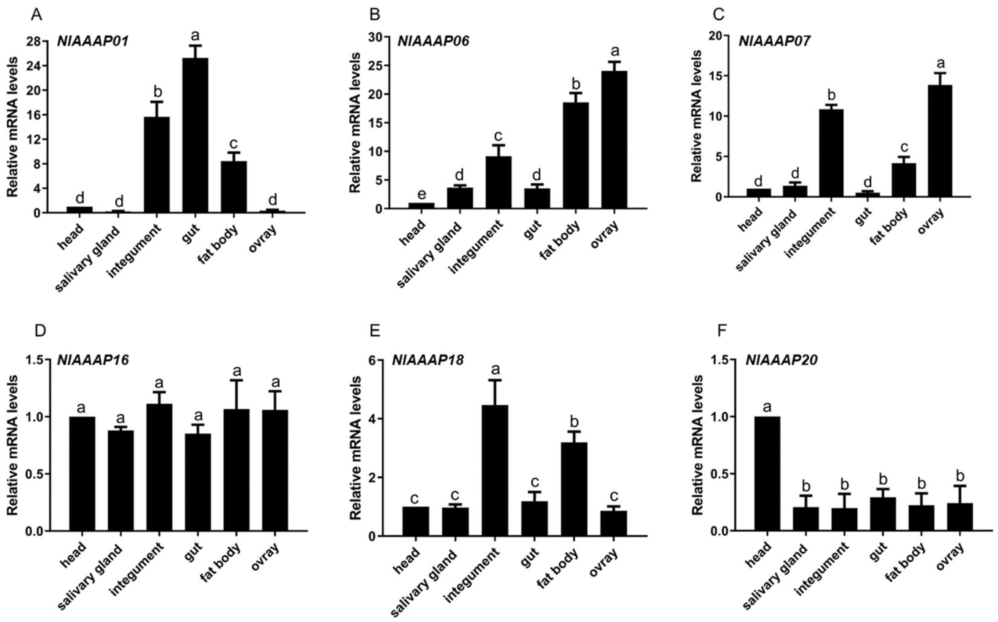

Figure S3: Tissue-specific expression profiles of some selected AAAP transporters. The mRNA levels of *NlAAAP01* (A), *NlAAAP06* (B), *NlAAAP07* (C), *NlAAAP16* (D), *NlAAAP18* (E), and *NlAAAP20* (F) in different tissues were determined by qPCR analysis. The mRNA level in the head was set to 1. All mRNA levels were normalized relative to those of *β-actin* in the corresponding samples. The data are represented as the mean ± SE (n = 3). Different low-case letters above the columns indicated significant differences (P < 0.05, Duncan's multiple range test).
