# Supplementary material for: Genome-Wide Analysis of the Amino Acid Auxin Permease (AAAP) Gene Family and Identification of an AAAP Gene Associated with the Growth and Reproduction of the Brown Planthopper, Nilaparvata lugens (Stål)
Source: Insects. 2021 Aug 18;12(8):746. doi: 10.3390/insects12080746 (PMC8397168; doi:10.3390/insects12080746)
Supplement: Supplementary file 1 [file insects-12-00746-s001.zip › Supplementary Tables.pdf]

Table S1. The predicted protein sequence of AAAP transporters identified in *Nilaparvata lugens* and *Helicoverpa armigera*.

Gene	Amino acid sequences
NlAAAP01<br>(*Nlug16008-TA*)	MEVSSEGKDNSSSCGTLPISARSTDKLELGKLEKNGKTQD<br>KNIRTMQLATTDLDALLHVAKSSLGTGLLAMPFAFKHAG<br>VIEGLISTIIVGLICAHGTHLLVKTSQRMCQTLKKTSLGYA<br>DTVEAVFSSSRHTRLQSKASLVRKTVNMFLFLTYYGAEM<br>SYVVFIAATLKEVTYHNLGYDMNIRLYMIMAIPPLLIIGTV<br>RSMKYLVPFSMLANMLLLSGVVFISYYVLQDLPPLSSRPN<br>YIPITKFPIFFSTVICGLEGIGTVTYHNLGYDMNIRLYMIM<br>AIPPLLIIGTVRSMKYLVPFSMLANMLLLSGVVFISYYVLQ<br>DLPPLSSRPNYIPITKFPIFFSTVICGLEGIGTILPVENSMRN<br>PHHFLGCPGVLNIAMSIVVSLFTTVGFFGYWKYGDAVRD<br>TITLSLPEGYVASTVKLLVALAIMFTYGLQMSAVMEVVW<br>SAIEKKFESKNLDVAYYSTRASFVIVTVLSSIAVPQLGPILS<br>LVGAVGFSTLGLFIPAAVDTILLSESHFSFTNWLVWKNMG<br>MMALAVFALITGSYLSIETIIVDYS
NlAAAP02<br>(*Nlug07328-TA*)	MASTINMKNSGALDIRNTGSTESFNTSSQIELAPASTLDQ<br>DQKKKKEVLQSRDHSKATSDFGALLHVIKSSLGAGILASP<br>DAFKHGGLIFSIFGTIFMGIVSTHCTHLVVKASQDLCFML<br>KKPKLCYPETAEGAFQEGAGKRLRRYARLFRKMVDFSQF<br>ITYYGINTAYVLIISSSAKEVVEYHFGVSYNIRWYSLASTL<br>LVVPIGCVKHMKYLVPFSAMANFLLGIGITISFYYIFQDLP<br>PIDSRPAIQSPSSISLFLSTVLLGMEGIGTIMPIENSMRHPSH<br>FLGCPGVLNFAMLVVVSLITLAESIKICVALAILFTYSLQLT<br>ASIEVLWDNVKHNFNKENQGKAYYVIRVLLIIGTVLLAIA<br>VPNLSPVISLIGAVGFAVLGITIPVIIDTILYWDEGFGMGNW<br>VLWKNGVLFLISLYAMIAGGITSKKISVTEKNGTNFSTSVN<br>VCDAKNAKQSGNGVFNEANTRTMHLATTDLDALLHVIK<br>SSLGTGLLAMPFAFKHAGIIEGLFATIFVGLLCSHGTHLLV<br>KVSQQMCTVLKKPSLTYSETVEAVFQTSPHGRLKAQSSL<br>VRKIVNAFLFLTYYGSNTAYIVIVAATLKEIAKDDLGYDY<br>NVRWYMLISTLPLLLIGTIRSMKYLVPFSMLANALLLTGIV<br>FIFYYILQDLPPITSRNEFKPISNFPIFFSTVICGLEGIGTILPV<br>ENSMKKPQHFLGCPGVLNIAMTIVVSLFTSVGFFGYWKY<br>GDNVRDSITLNLPPGYIGSTVKLLVALAILFTFGLQMTAV<br>MEVVWFSAEKKFTPPNRDIAYYVIRGILVVITVLCAVAVP<br>QLGPVISLVGAVGFSTLGLFIPAAVDLICASDRGINWKSFS<br>VWKNLLMMALSIFALITGSFTSMRTIITQYT
NlAAAP03<br>(*Nlug13074-TA*)	TFTEIMDQNGLDNKGFEKGYENGMEKTPSILTLQNNDNN<br>FDTSSRLKLTKDSFPEKNGKKYPSKEESYDPFSNRDKSHA<br>TSSMGAFVHMVKSSLGTGILAMPGAFKNGGLIFGVIGTLL<br>TGLICTHCVHMMVYCSQILSIRVKRPTLSFAETAEVSFQTG

	PPKYRFLASFAREMINVTLFCTYFFGNTVYIIFIARTFEQVI EGHAHFGWDVRVYIVLLAFVLVPLGIIRTLKYLVPFSAIAT AFISFGLALTLYYTFSEMPSDVSNRDFIGTPLKIPLFFSTVL FAMEGIGTVLPIENSMNKPQQFLGCPGVLNGAMSIVVILY VVVGFFGYLRFGNATQATVTLNLPDSALAESVKILVALSV LLTYGLQFTVPSEIVWKRLGPRVSEDKQDMGYYIMRGSM IIGTVIVAIMLPSLAPIISLVGAICFSILGLFCPAVIEIITFWEE GYGKYQWKFYKNVFLIFLSIFSLVSGSYASILEILDAIKHG EL
NlAAAP04 (*Nlug04460-TA*)	MGTNGHVMTLANSIIGVSVLAMPFCFKQCGIMLSILVLL MSGVMTRLACHYLLKSAINARRRNFEILAFHTFGPTGKLI VELSIIGFMLGTCIAYFVVMGDLGPAIIGPVFNIDSTPSLRP SVLMGLGLLVVLPLGLLKNVDSLSTICTATIAFYLCLVLK VMAEATTHLLSWDWVDKVNLWRPAGISQCIPIFAMALSC QTQLFEVFDSVPNTPLDKMNAVVRSAVNMCTAVYISVGF FGYIAYCTQNFSGNLLMSFSPTVMSEIVKLGFVLSVAVSFP LVIFPCRASLHSLLFRRHGHTPHHELLSGGGNYIPELRFKC LTVCIVLVSLGTGLLIPSIEVVLGLVGSTIGIVICIIFPALTFIC LSNKKNNERLLAQMLIGIGLFIMVLGTYSNLFATEEAITKP DVDKQGTSIDLNQAGSPQLPEEAAAIIKEKSEGVAVAGIPE IKQQANDVLLQIKKVIDAEDSDKRKEPPVPVAPGDNIVKD SNVNSPISSAVKQMKEEMEKENEDMKPTEKGEGIQKETNI LKEPIKKNDVLIKEDQLKKLYKIEVNSPIKSETLEKSPNAE VKEIKKPKRHKEDVLKIMAKVGNSDRDQEKNVPPYENN KNNIDKEDEISNPKVNNIIKLDDTAKQNDEKSIKLSDTLK VPRQEIELKTASDTDLGNKKLGDNQEKNGVDITPDLEKNI IKGVPLPIAVRNNMNKNSVELDKPFLDIEADKQKIGGVRD ILSEKEKFVKEEREKRDLETVEKEDMNNFEKDVVPEKPE VESTIDKQNVIEKEKSNSIKGEVEKPVEHKDVVPSSGSHL GTVDSEAKCSKDSKNQFKEDENGLQASILKMELKKIPQE SEKDMEMPVTDKQVLQTSVEPLIKTPKFMSPPETVLNAV ELKPEELIRTAQDVKPMSRDLKSVNNDQGNTKNTRQQDR ITEIT
NlAAAP05 (*Nlug00688-TA*)	MTEPPKPDEKSYILNAHKAVLEQQSSGESPEGRSGTHQDT EQLFVDKKERTTGLGFASFNYINSILGSGVIGIPFALHQAG FGFGFLLLGFMALVTDYSLILMVRSAHISGSFSYQGLMEA AYGQPGFYLLTFLQFTYPFIAMVSYNVVVGDTVTKVLIRI FDLRPWSLLARRDFVVLLATVFVTVPLCLLKDMARLAKA SLLSLFFVVFILVAIVVRLFTFSSVVPTTSNAWQLYNWGVI PAIGIMAFAFMCHHNVFLLYGSIEQADQKSWETVTHYSVF ASFIVACLFGLTGYATFTGFVQGDLLENYCRDDDLMNMA RVAFSASILLTYPIECMVTRAVVDQVVNITNATMRHYGVT LIIISVTYFLSVTTDCLALVLELNGVMSAVPLAFILPAASYL KLEEGSVLSRKKLPALGLALFGTVVAVMGAFMIFRHFSSV

	ERCAHGRVMEYCLPSFANKTHSF
NlAAAP06 (*Nlug19955-TA*)	VPPLAAQLDNSDSNVAIPIGPEVSDPTPPVGGKASKAVGT PNSDYNPLNNRDLEHPTSNLDTMIHLLKGNIGTGILAMPD AFRNAGLVVGTVGTLLMGIICTHCMHMLVSCSHELCIRH QIPALGFAEVADHAFRSGPNRLRNLASYARKIVNTFLVITQ LGFCCVYFVFVAANLHEVIKHYYMSISVPWLLLILLIPMIL LNWVKNLKYLTPVSLVAALLTGTGIVISFMYMLQGLPRTS SVNAFASWHQLPLYFGTAIYAFEGIGVVLPLENNMKTPQD FGGLAGVLNTSMVIVACLYTAVGFFGYLKYGDRAVLGSIT LYLPQRDLLAQLVRLMMALAIFLSYCLQFYVPMSIIWPPI KRRINDESYQKVAELSLRTFLVCLTFLLAVAVPNLSAVISLV GAVSSSMLALIFPPIIEMVTFWDKGLSPALIIKDIFILLFGIF GFIFGTYVSMYNIVTK
NlAAAP07 (*Nlug15359-TA*)	MYQIIGLNLGTGNVKCAHELYYRTRVTSMSFADVGEIAFA NGPPWARRFSGLARVSIIVGLFAAYFATCSVYTVIIATNFQ QVIEFYTGDDEATSGERIRLYIAALMIPLVLISWVPNLKKL APISMVANAFMGVGLLITFYYLSIDLPSVAERAYVAPFEA MPQFFSITIFAMEAIGVVMPLENAMKTPQHFIGICGVLNQ GMGGVTMIYIFLGFLGYLKYGAETKGSITLNLPIEDYAAQ AVKILVGLAVFCTYGLQYFVCLEIVWNSLKDRFQKNSAL AEYVVRTILTVAAVLLAVAVPTIGPFLGLIGALCFSLLGLIIP VFIEFVTYWEKGFGPGNWIVWKNILVLICGFLALVFGSYT SLLEIINVKDLPSPLETFTNESSLLGNETMTHLNATLQQLG EVVNSTLEAAVDKLT
NlAAAP08 (*Nlug12813-TA*)	MTASSPKELQQNRSLEKDAELEKGKGGGHGLPPNKHPTS FIETLVHLLKGFVGSGVFALGEGFKNVGLILGTVLTVFIGI ACVYCEHILLKCSTTLKKRRKLSYYPDFAETVEMCFEEGP QSLRKFAKFTGSITYFMIIFMQFGFCCVYILFISNTSKYISD SYGLNLDLRVHMCIMMVPLILSSLVRSLKYIVPISLTANLC MIFGIASTMYIVMQDLPPVSSRRYIGDLGNVPLFFGTAVYS FEGIGLVLPLKREMKNPKHFDRPLGVLNLGLVIIVSIFLMM GFFSYLKYGDDIQASVTLNLPEKLAITDGESGNRNWNIVH ICTHVLCANRTDLAEDRSQVRSSEQATPLRTHISNIHGFCH KLSQMVRVAIGIGILFTYALMFYVPIELIWPRIAARFGPLSK PLLSELIFRIFMVFVTIFIAECIPHLGLFISLIGAFCSAFLSLV VPTVCDLILRWPSTFGTMKWRLALDIIALFLSVIGVTSGT YYSLEAIVLAFIGESS
NlAAAP09 (*Nlug01999-TA*)	MVKENTLNCQNLKLQLRYVPVALGGSSASTCDKQQLSN NREFKRKVIALSRPPGTPVTPKSPHPTDCLPQCRDANPGIS LFEKIWKTPIARIGYEYYACIPMRLTRRPKSGGGWGGDRQ LKQCGSDHCDGWHGNAKANALHTPQYKQPPLQYYETFR KIECTNNLIQLYVFRFTVWNTMVGTSLLAMPWGMWESG PLLGTLLCLVMASVCLYTAYLNVSIYSKNGKPGEGAELFV LIKDLIGPWAEIIAKLFSVLVLAGAAIIYWILLSNFFYHNVY

	FIFYYLRNDSNMQPTWSTTVLCPNHENFTSAESRAEEAHDTFNSIWNLHRTVPLYLILILFPVLNFKSPTFFTKFTSLGTMAVMYIMFFALVKFFVFGVNVSHDPESEYYTSFAHWTTFPKMSGMLTLSLFIHNIIINLMKCNEKQENNKRDLSIAYILVCTTYAFVGLVIYVSFPAAKSCIQDNILNNFLTGDFMTLFARFFLFFQLITVYPLILFMLRNQVLTMIFQSNYPSVSSVVGFNAVIVSLCVFFAIVFPQIGHIIRFTGAIAGFIHVYSMPCLLKIMSEKADGSLTTTSMIIHVAIPILGFINIIAQFIV
NlAAAP10 (*Nlug07975-TA*)	MSKGDSIGLQKVNGNKGSNYSVESGSPGPPYDPHAHRELANPTTDRETLIHILKGSLGTGILAMPNAFYNSGLIVGTVATFVIGFICTYCLHVLYYFCFRPIVDGFLIIYQLGICVCYIMFVAQSIKQVADIYATPLDVRLHMLIILVPLMLITYIRNLKLLAPFSQLANIITFIGIGITLYYIFDDLPPVNSVPYVGVLRNFVLYMGTTLFALEAVGVILALENNMKTPKSFGGYTGVLNQGMIVIVILYVLVGFFGYVKYGKGALGSVTLNLPSTDILAQVVKLIFAVAIFITYALQCYVPVDIVWNTYMKKNISTKHLFWEYVLRTAAVLITFVLAVAVPRLELFISXFGAFCLSALGIAFPAIIEICVMWPDNLGTGYYILFRNFLLILFGIAGLLVGTYTSISDIITSFSK
NlAAAP11 (*Nlug10573-TA*)	MSCQPSLLNITNESFKNELNKSAVADPGLNLNIFVIGSDIKDGNRSTLKLVEKSKEELNEEEYDPFSFRQVQHPTTYGETLVNFLKSLVGTGVLAMPYGFQNTGMIIGILGTCIIGFISAYCGNTLVRCSHILLKRRKIPILDFAGVAEQAFLSGPQRFRKYSMFVGKFIDLFIVLGTIGGNGCYIVFIASNLKQLLDPMVGEHSSRIYMVAMLPVILVMNLVRNLKYIAPFSMFANAVLGSGLVIIFYYIVQDLPAVSTMPLYASASQLPLFFGTAVFAYGMIGVVLPLENDMKKPTQFLGVGGVLSIGVCFTAVLYTTMGFLGYLKYGEKTEASITLNLPLDEPIAKSVKLLYSVAVLFTYPMASYPAMQILQSYSAKSFKSLSVLYFFRVLMDVLTVLLAILIPNLGAFVSLVGAVSSCLFGLIFPPVIELLTYYEDFNYGRFYSRIWKNIFIIIFGALGFITGTYFSILDIINFDK
NlAAAP12 (*Nlug01820-TA*)	MALGPHKTTEPATKMTRDTLSIALHNSGKNDKSEKNGLDNPALVMGSKTSINPPEMYSAPPYSEKGMSKDVLQSVYVVPANGTKTEKEEEYDPYLNRNVKHPTTYWETLIHMWKASLGTGILAMPNAFHNAGFAVGTIGTLVIGFLCSYCIHILISTQYELCRRRKTPSMTYPATAEAAFQDGPTWTRRIAPYAPGICNAFLLAYQLGSCCIYVVFVASNIKSVMDDYIEPMDVRVYMVILLVPLILINWVRNLKYLAPFSSLANILTVISFAITAFYVFQDLPDLSTRAAVGSFKGMPLFFGTVLFAMEAIGVVMDDYIEPMDVRVYMVILLVPLILINWVRNLKYLAPFSSLANILTVISFAITAFYVFQDLPDLSTRAAVGSFKGMPLFFGTVLFAMEAIGVVMPLENEMDNPKRFGSAFGVLNCAMLPITILYTIVGFFGYLKYGENAAGSITLNLPGDQLLAQSVKLMLALSIFVTHALACYVAFDITWNQYMGPRVESKKMFWEFFCRTMLV

	VVTFGFAVAIPNLELFISLIGALCLSTMGLSLPAVINMLTFW<br>DQYRGVGFILFISKNMCIILISVLGFAVGTSTSLHEIIEKFFH
NlAAAP13<br>(*Nlug01818-TA*)	MVQKISSDGAPLHKSTTISVAVSGRNGHPMISEYDQKKGS<br>SRTELADLVIVKYKCESNGVPMKLTNGSTLPLVPGSSKEA<br>EEGEGYNPFAHRVLEHPTTDLETLIHLLKGSLGTGILAMP<br>LAFANAGLLFGLIATFLIGFLCTYCIHVLVNCAHILLKRMK<br>IPSLGFADVAEVAFLAGPQSTRKFAGFARGMINSFLVLDLL<br>GCCCVYIVFVAENLKQVVDTKAGVDWDLRFYMILILPFL<br>LLVNLVRNLKYLAPFSMVANIFIGVGMGITFYYIFDDLPPV<br>SSQPQFSSWHQLPLFFGTAIFALEGIGVVMPLENNMKTPA<br>HFIGCPSVLNIGMFLVILLYTGVGFFGYLKYGADTAPSITL<br>NLPRHLALAQSVKTMIAVAIFLTYALQFYVPMEIIWKNTK<br>HRFTSHPIAAEYAIRISLVCLTSKYIRYSSFASDIM
NlAAAP14<br>(*Nlug01815-TA*)	MTTKSDRVSYHDDNKTDTPAVPEKIPLLLPTIKQAGIGDIE<br>TLVHLLKSSLGTGILAMPMAFKNAGLIFGLLGNIFHRIHL<br>HLLCPHVKCAQILCERKTIQKIGFQKVVELSFLEGPKATT<br>KFSTAAGKTVMAALLMELLGCCCAYVVFVAKSMKQLVD<br>HYGGHEMDVRFYMLLLVPCLITTNMGKSLRFITPISVISNS<br>LFMGGVGVSYYYIFDDLPSVRERPWAVSWHHWPKFFGT<br>AIFAMEGIGTIMPIENSMKTPKHFIGCPGVLNIGMIILVTLY<br>SITGFFGFLKYGAATDDSITFNLPTGAPLAQVLKIVIALGIF<br>LSYSLLFFASIDLLRDNVLQRYAENSIRIWLIVLTVVIAALF<br>PNLGPFLTLVGALSLSIVGLIFPPICEVLVYWHSPGLGRFN<br>WRLWKNILIVCFGIVGLITGTMTSISEFARAYRR
NlAAAP15<br>(*Nlug01817-TA*)	MEISEGEKGRDYFGESGLEKVCMLEYSDVIDPPELIPKPM<br>KDIAWRNGISAIACSGQEKKAATIAKNVISQKDQEAAFYD<br>PFMNRNVAHPTSDFETFVHLLKGSLGTGMLAMPMAFKN<br>SGLFVGLVGSIVVGYVCTYCVHMFVRASHVLCHRHQLPA<br>IGFQKIVELSFLEGPECLKKFSKAAGYVINFGLLTELLGVC<br>SAYVLFVAKSMKQIVEHYWHVDMSVQVYMAIMLPVLITI<br>NLTRNLRHLTPLSMISNVLFLLGVFVSFYYIFEDLPPISSRP<br>ASFSSWGQLPLFFGTTIFALEGIGVVMPLENNMKNPKHFI<br>SCPGVLHIGMVFLIILYAITGFFGYLKYGDKVEASITLNLP<br>VETMLGQFLKTIVAIAVFLTYFLLFYAAMDLLGIVNGRRF<br>KTRPLLKENIVRILTVIGTVVVAGLFPNLGPFLSLIGAFSLC<br>VVGLIFPPIIDTLIYYREGLGRLKWRLWKNGVILACGVLG<br>LVTGTMISLQEFVESMGGA
NlAAAP16<br>(*Nlug01797-TA*)	MADSGYEPGDHRQHLKHPTNYMETLIHFYKACIGTGIMA<br>MPYAFKEVGWASGLAITIVIGSLCVYMTNRLAISSRRMCI<br>LEQKPTMSYVNTANACLKYGPELTKNFKYFGSTANMFLI<br>LYQFGCSCIYVVFVSGNLKHVWDYCYPETNPDIRLFMLIV<br>MIPQCIVGLVDNYKHLSYFSLLADICTISGFVLMFACYLLV<br>DTKSLSEISPSNNGVYRFPMFVATVFFSMEAVAIILALENE<br>MKTPKHYTGYFGIVNMAMIPAVVLYTAFGFCGYLKYGED

	VKSSATFNLPVGDRIAQVILFSMSFAIFVTYHLSVFVTYDL<br>IWKTYLGGPDESKNTETHVEINFFRHYMIRLAIILGTNLCA<br>MGIPNLSLFIVLLGALTLSVTGFAFIVMMWVCIYWNQYSG<br>VKFAIFIAYNAFLLFLAGCVAVFGLGQAIYDIAIFYDGEETF<br>D
NlAAAP17<br>(*Nlug07143-TA*)	MERDEIKVNLNGDSYSTEKCMITHQSELPLLMSSENDGK<br>LQKEEEGDDLHPGVKHPTSYVETLINLIKGNTGAGMFAM<br>GDAFKNAGLLVGPILTVILGIICVYGNHILVNCSVAIREKK<br>KMKKPLAFAETVQICFEEGPKFARPWANTVRICTYVFITIT<br>QLGFCSVYFVFISSTLVKILVPYGINIAAHTYMTMILLPILA<br>TALIRNLKFLTPISSLANLSLGSGVLLTLFFASRDLPPLATR<br>RMAADWKQLPLFFGTTVYAFEGISLVLPLQNEMKNAKKF<br>DTKFGVLNMGMTVVCAILISIGFVGYLKYGDDVKGSLTL<br>NLEGTGLFSEIVQLNICFGILLSYTLQFYVPIKIIWPHVERK<br>FAPMKHPVLCEKLFRTAFVIGTYFVALTVPHLGHFISLIGA<br>VCGTTLALIFPPICDLVIRWPSDFGKLKWRLILDIFSLLVAA<br>IGLVTGVYYSLEAIFDAYSNTT
NlAAAP18<br>(*Nlug10388-TA*)	MPDEKLSVNSSMGRSVNYQSTSSSREPIWTRVANDPLSGS<br>CSGYESNESYDRSLTSCTSSEPCKGKGLNVFNAAVFVAGE<br>MAGSGILALPKAVVDCGWIGLVLVVVFCLNACYGGCRL<br>GKCWTILEERYPEHRASSRNPYSTIAYRAYGRWCSLLVSG<br>CIQVTLFGAGVVYLLLAAQIFQELLRTIVPEIGYCMWFLIF<br>AVLTIPVMWLGSPKDFSFAGIGALLTTILSCFLIIAQIATEGL<br>ELKHPPQHAPHTFKEFFLAFGVILFAYGGASTFPTIQNDMT<br>HREKFPKSVAIGFAVIFLLYLPVSFGGYFVYGDAVNSNILL<br>SLQRGPFELVANILMAMHLLLAFFIVVNPVSQEIEEIFDIPH<br>SFCWKRCIIRTLMVVAMIVVGETIPKFGMILALVGGSTITL<br>TTFVLPSLLYMKLCDQKSVDWPDRYIPLYERVYMWELIVI<br>GLLGGAASTYSAMTAIFSADALTKPCYWPDVK
NlAAAP19<br>(*Nlug10180-TA*)	MEETYSLWTGLIYVFNLIVGTGALTLPAAVLKAGWIVGIA<br>MLCFLAIVSYITLTFVVEAMATANAITQWYKMKRLKRVS<br>QNLCMVINQGIGDDDDDHDNVDDDDDDVERINDDNDEN<br>DSMLSSEDAPLYSAGTLRNFPPMPVTFFSIDEKVEMGQM<br>ARLFFPKVGQFLFYLTFCLYLFGDLSIYSAAISKSLVDVVC<br>EADRNSSLLKDNEPCFENYNITQKTAYRLFLSAFLVVFGP<br>FVFFNVQKTKYLQILTSLTRWLAFTSMIALAIRRLLSPSLP<br>HGDPTPAHLHGVPTLFGACIYSFMCHHSLPGLLAPIHATS<br>KRRLLPGLAADYLLILLFYLTLVLTGIFAFPDLQPLYTLNFT<br>PRPGDSGYLRAVDYFLALFPVFTLSTSFPVIAVTLRSNLQA<br>LLSPSTPYCIRQIIVPAFGVIAPVLLAVVVDDVNTLVGFTGS<br>YAGTGIQYLIPAFLLISAKSAVPPELARYRNPFASPFRSTTW<br>LVLVICWSIVCITFVSINLIKY
NlAAAP20<br>(*Nlug02695-TA*)	MAAHLFNFQLIINEKPKRGMMDEERVPLLWKAPTTEKQY<br>NEGSERREGSKSSSIQGLSLLSATLCIVDLFGVFPVVTMPR

	IIINCGWFGFPLVLGVLILQVYTALLLGRCWIMAERLQPSI VKQSRRPYAALAEMTYGESFARLVTFILDITVFGAGVPNLI VVVSSSMTVTTVSLLTWHCFMASDVATPPPLPSISWEAIAI GYGVLAFQFDIHPTILTIQVDMADKGKIGCAVIIGFLVTGT LFLTTSVLGAIYYSTELDYNVMQTLQPSLVMDINVLLVIL QICLSTVIGATPLFQDLEDKLGVDKEFNLKRILLRSGLVML SLALAEMVPRFDLIMGLIGGTLMGPLMFVLPPLFYARLRA MEPQPQLRPVATRNTETATIITVAFAPQPPDQIEYSMKQTH YTNPRRSQKPRRSKQTFEGKSSYNLIPKEKYLDDLPSPTS QPNFFTKIKSNFLNLATLDDYPKIGPMTAWERLKTAVVVI AGVSATLISTYYSVRGTIRYARFAPPCLFNVTAATLAIED
NlAAAP21 (*Nlug04947-TA*)	MFKWKYKRIYQMIEYKHYLKKKIQKDTVFKSVTIKLNPG KNVNLGDSENCFRKAITYSCAYLQESRAADKNLVTYLQS TQDTGSYQMSRGGTFSMGESTDDFGGEFGGEGKSRHKIN EWQAAWNVTNAIQGMFIVSLPFAVLRGGYWAIFAMIGIA YICCYTGKILVECLYELDTSTGQRIRVRDSYVGIARECFGP VWGARAVNCAQMIELLMTCILYVVVCGDLMGGTFPDGSI DTRSWMMLTGILLIPLGFLKSLHHVSLLSFWCTMAHLVIN AVILGYCLLEIGDWGWSKVKWTLDLENFPISLGVIVFSYT SQIFLPSLEGNLIDRSKFSWMLDWSHIAAAIFKSLFGYVCF LTFQNDTQQVITNNLPSAGLRGLVNFFLVIKAILSYPLPYY AACELLEKSFFRGKPETPFPSIWHLDGELKVWGLAFRIGI VFFTIMMAIFIPHFAILMGFIGSFTGTMLSFIWPCYFHLKLK GDSLDFGTVAYDCFVIFLGVVFGVIGVYDSGSALIKAFEIG LPF
HaAAAP01 (*rna-XM_021337951.1*)	MPRRFWGLRRLIRMPLTEAGDQYSVWVGLIYVFNLIVGT GALTLPAAFARAGWGLSTISLMFLAFMSFLNATYVIETMA CANAVFKWKRLQTIKRESIQDHESDEDPSTSQNNHGDLE EPLVCGDNIPSRYYSLDRRVELGEMANLFFNKIGRTTFYC SLCIYLYGDLSIYSAAVAKSIMDVVCTPIPPNMTDSKVWD ELPCFNISSTDRTGYTRFDTYRVALLTFVAAMGPFVFFNV QKTKYLQLFTSGMRWLAFTIMISMAIHLLVGRGAQGHPP AFDVTGLPTLFGACVYSFMCHHSLPGLIAPIRGKSRLGLH LFFDYAIIAMFYLLLAFTGAFAFAHLNDLYTLNFIPADNDN IFLEVVEYFLALFPVFTLSTSFPIIAITLRNNLQSLFLDTTRL ESYNFVLRKVLFPIITVVPPVLLTYFLEDISILIEFTGSYAGT GIQYFIPTFLVVSARRHCTNLLGLGVVNKYKSPFSHVAWA IFVLLWSFMCIILVSVNIFEKNL
HaAAAP02 (*rna-XM_021327065.1*)	MNLNMENGSVRSSIENVPSTTQSTITLDRKDDDKDEKND YNPFEHRDVVHPTSTLGAFFHLLKSSLGSGLLAMPAAFR NTGLIPGCIGTVLVGVIATHCVHILVSTSRAVSKNCRAPSL SYTDTCEEVFKQGPKKLQQYSKHIRHFVDAAMAGVCLG GTSVYVIFIASSLKDICDHFVPSKKFEVEVYCGILLLPLILIT QIRHLKFLVPFSVLANVCLVVTFGITCFYTFTDLKPLENIE

	MVASVEKWPLFLSTAIFAMEGINVVMPVENEMAKPQHFL<br>GCPSVLNVTMVFVTILYGVVGIFGYMKYGEGVLGSITLN<br>LPEGENLALTAKILVAVAVFFTYCLQMYAPMDIIWTRIKCR<br>ISKNYFNFSQITLRTLSVTLTVVLAVAVPDLELLIGLVGAIF<br>FSTLGLLIPVVVQTVHLWEKGLGKFSYILWKNALLLIFYFI<br>VLVSGCYTAVSEIILKFR
HaAAAP03<br>(*rna-*<br>*XM_021327047.1*)	MAKAKESMEIDNFKSTADLTSNPGFQSSLSIASKNVDEKP<br>YNPFEHRTVEHPNSTIGSIVHLLKCCLGSGILAMPAAFKNS<br>GLIVGAIGTLIAGFVCTHTVHILVKTSQEVCVDAKKPSLSF<br>AETCGAAFTYGPKKLQSWGNSIKVIVDYSMVVTYFSVLC<br>VYVVFIGSSMKEVMDVYMPDNPISIQACCALTLVPLVLIC<br>QIRNLKYLVPFSALANILILVVFVITLYYVFVDLPPLKERE<br>MVASITQWPLFLSTVIFAMEGIGVVMPVENEMAKPQQFL<br>GCPGVLNVAMVIVISLYGFVGFFGYVQYGDMVKGSITLN<br>LPEEDVIAQVAKALMAFVIFLSFALQFYVPMEMITRKRKG<br>KESKYENFVQIAIRTVMVTVAVAIAAAFPNLELVISFVGAV<br>FFSTLGLLIPAVVDTVYHWDSGLGPFNYVLWKNLLIGIISII<br>ALVSGAYVSVQGMIEDFGSSSHEAFDNATLT
HaAAAP04<br>(*rna-*<br>*XM_021327029.1*)	MGNDEKKGSIVLENFNSTANLASNPGFQSTLSLGSKEVIN<br>EKAYNPFEHRKVEHPNSTIGSLVHLLKSSLGSGILAMPAAF<br>KNAGLAAGAIGTLVVGFICTHCVYVLVKTSQEVCVDAKK<br>PSMGFAETCGAAFEFGPKRLRPWANFARTFVDYAMTCTY<br>LAALCVYIVFIAENFKEVLDEYIPEYKLSVEAYCALTLVPL<br>VLICQIRNLKWLVPFSAIANVFLVICFAITMYYIFSDLPKPE<br>GRVVVASVTQWPLFISTVIFAMEGIGVVMPVENEMAKPQ<br>QFLGCPGVLNVAMTIVISLYGIVGFFGYMKYGDDVRGSV<br>TLNLPQDEVLAQSAKILMALAILFTYSLQFYVPMEMIWR<br>QLHNKVSVRYHNITQIAIRTTAVVGSVALAAAFPDLELFIN<br>LSGAIFLSTLGLLTPAIIDTVHKWDRGLGPFKWILWKNIFI<br>AMISLVALFAGSYTSIRSMVDKFYETPVLEIVANVTSVSM<br>NDTLSV
HaAAAP05<br>(*rna-*<br>*XM_021327054.1*)	MSTRYGRALKMFGDVCEQITAFCGLGIELAYNGSKEDLE<br>PYIPSQHRPIASNTSSFGALAHLLKASLGSGVLAMPLAFK<br>NAGLAVGSVGTLIIGLICCHVIHVLVKTSQKLCVEVRKPA<br>LGYADTCDLVFQHGPKPVRKVAPFIRELADWALAVTHMG<br>ACCVYVVVVIAESFRQVSMVYGGPDWSVTVYCALSLVVL<br>LPLTQITKLKYLVPFSALANFVWLGSICICLYYCLKDHPKV<br>SERKLATSISGIPTFISTSLFAMEGIGVVMPIENEMRKPQDF<br>LGCPGVLNAAFTVIAVMYAFVGFVGYLRFGDEVRGSLTL<br>NLPQEEVLAQTAKLLVGCVLLLSFTLVYYVPIDVIWRRLE<br>HRIPLSGRRWGLAGVRFVGCLLLVGIAAAVPKLELFMELV<br>GAVCLSVMGLLLPAIVETVFRWNRDIVPCSFVVVWKNVIIG<br>FFSIIAMGSGVTYAILAIVNTM
HaAAAP06	MRKSSLAQDTSSRRLPGQEARRLSGQEARRLSGQEARRL

(*rna-XM_021327435.1*)	SGQETRRLSGQESRRLFGDEPLSTIDFRRTRLPSMRPDLDD<br>YDPRDHRKPVHKIKVWMAYSNLFRATTGVGMLAMPFIIS<br>TTGILLGPILCLLTGILMIHAHSLLLDTLYEVARQLKMPYIS<br>YRYAFRLALLHGPPIFHGIANYGPIIIATCLFVSQLGICSVIV<br>IFTTDCLRDLMDWQASQTALISLIFPYFVMEFFMRNFTIVS<br>YVAVIGTILNLLGIALVFEQLIEEAQGETIRFSAMLNYVLFS<br>FGVMQFNLCAIGVVMAVDKHLENPRVMRARFGVINVGI<br>MIPTIITLIFGTVGYWGFGTMEENILRCLPFQERTSLATVCI<br>YMLAMILTYPLQSAPAIHAILEVVKYYEKPGWPQPSEDTL<br>FAIENISKPIFVVLSFLICYIVPFQAPFLAFVGNLCASMLAL<br>VFPAVMDLCLRYPHYYGKHNLHLFKDMAMIFVGIASILF<br>GCIFTTELINIRLKTKYSPNSYGFF
HaAAAP07 (*rna-XM_021328263.1*)	MNVEKAEEKREYNPFEHRQVEKPTSDIRSCANLIKSSLGS<br>GLLAGPLAFANAGWGVGLFGTLLVGVICGHCIHILVRTSQ<br>KCCVLDKKPSLGYAETCKSAFMNGPKGIRRFANIASVFAE<br>FALFCTYTGVCCIYTVLISDSVKQLVDRYAPSTILPVEYYC<br>LIFLVPIILLCQIKYLKFLAIFSGLANILLVTVYIVCLYYIFG<br>GDITFAGKHAAGNPARYPAFLSTVIFAMEGVGVVMPIENT<br>MKKPQNFLGCPSVLVVAMSAIVFLYGTLGMFGYLRYGDI<br>LRGSITLNLPTDEWPAILAKVSIALSIFLTYPLQFYVVIDIFT<br>RYTQHRIKENYRQITQVVARTIGVCCCVGIGMALPMLEQI<br>LNIVGALFYSILGLIIPSVIETVFRWENLGKWNWILWKNTF<br>IALFGLLSLVSGCTVTIMDIIEILNRKTE
HaAAAP08 (*rna-XM_021328232.1*)	MIEKKEKSKNVEDYNPFEHRDVVKPNSDMRSLANLIKAS<br>LGSGLLAMPLAFANAGWGVGIVGTVIVAFICGHCVHIFVE<br>TSRGCCRAERKPLLTYSETCGAAFANGPKCVRPFANFAH<br>MFAEFSLCFTYLGVCCIFTLLIATSIKQLFDTYLSTPPIPVG<br>QYCLFLLLPLCLLCQIRHLKWLAPFSLIANVLLFATFAICL<br>YYIFREPLTFSDKQIVGDMSRLPAFLSTVIFAMEGIGVVMP<br>VENTMKKPQHFLGCPSVMVIAMTLVMLLYIMLGVFGYL<br>RYGEALRGTITLNLPMDAWPAICAKVFISITIFLTYPLHFFV<br>VIDIFTRYVEPRVQKKYHNVMQICVRIFIVCFCGGIGIALP<br>LLEQIINLVGALFYSILGLIIPGIVETVFRWEDLGKYNWILY<br>KNILIVFCGMFSLVSGCTVTISDAIEMIHKQME
HaAAAP09 (*rna-XM_021328152.1*)	MTNSYNMKEFSSTAVITENGLYPSTISISTINTKCKDNDLE<br>CNAYDPFQNRKLEHPNSDMRSFANLLKSSLGSGILAMPA<br>AFKNAGTLVGIVGTIILGYICTHCVYLLVKTSQEVSRVTRV<br>PSLGYAETVEAVFATGPPPLRKISRGARIFIDWAMAFTILG<br>ACAVYVILLVESVQQIVDFYFVDNEISKTMYCLMFLVPILI<br>FTQIKNLKYLAPFSGFANVLLVLTFLICLYYICSDFPPISDK<br>PMAVDIGKLPLFIGTVIFAMEGIGVVLPVENTMAKPNHFL<br>GCPGVLNITMSVVVLLYMFMGFLGYVRYGDLAQGSITLN<br>LDTSEIPALTAKVFIIFAIFFTYTLQFYVPMEIVWRNTKGYV<br>TQKYHNIAQGVIRAVFAVLTVIAAATLPRLEQVIGLEGAFF

	YSFLGLIAPSLMDVIFKWNRGLGKYNWILVKDVILIAFGS FVLVAGVTQSIREIIKTN
HaAAAP10 (*rna-XM_021328488.1*)	MGTTGQSITLANSIIGVGILAMPFCFQQCGVLLATLILLFM GLVSRLCCHFLLKSALLARRRNFEFLAFHVFGQAGKIAVE VGIIGFLMGTCIAYFVVVGDLGPQIIAKMFNINQSDILRTSI MVIVSIVCVLPLGLLRNVDSLSNVSAATIAFYFCLVMKVIF EAAGVMLTSDWNQRVEMWRPAGLLQCVPIFSMALFCQT QLFEIFESLPTLSLEKMNIVTKXXXXXCTAVYFTLGMFGY VAFASRDISGNILMSLSPTMASDVIKLGFVMSLAFSFPLIIF PCRASLYSFLYKKVHSSHHDHIINHSIPETTFRCITVGIVGV EIKKDIRKDSEIQPPNPVPPESQSNEKLPQTKKIIKTEEKIIL KPEIKEALDKVSETHPRNLKNKLESKLANGISNGEAQKTN EVKPQSNEDKIDMIKQQKLIETIKQHGEEQKELIKEQKEIL DEILKTKKELQQNKNEAEGAIEAKKIAVESIKQIADMAIKS IGGVSEKPDVKDEVKAERLEKLTNDAVQEIAKKAVETIEA IQEIKEKPEQPIAAPQNNLPNVQVQNIPAQNVPAQNVQPAI PVNPQVIQGANDLVKPKDQAPNNVAANVLPQVNGAVVN QNGAAVQNNANQQLKREVKPANEIAKENAQVPNIQPKIP ENNAQNQPKVDNVNKPVLNAEVGAEKPHSHSPDEPQSY KQGEDTQAKVPENKVVQNVPISIVNELANNQMKINRQPA AIQPNKDDIKVNVPPKVQNNGAKPNLQANVPPPNSNLQQ IVNQAHARQKREVVDCTKKVTLKPEDKAICRNLKDGNK DSQEILKSVDLNEVALPKSLPLDVNVLHHISRALKSFDKD ER
HaAAAP11 (*rna-XM_021329232.1*)	MSKKHLHNQAAIPLAPAVFKKPQMRPMIVEYDPKKKGV KNDLSDVVMVKYKVNPNEIPVEQEAGSTLPLMEIPGRDIE ADEDYNPFEHRKLAHPTSDMDTLIHLLKGSLGSGILAMP MAFRNAGLYFGLIATFAIGGICTYCVHVLVKTAHELCRRL QKPSLGFAETAEAAFLSGPPAVHKFSRLAKAMINWFLVID LLGCCCVYIVFVAKNVKQVVDFYATGSDWYDVDVRIYM AVLLPLLILMNLIRNLKYLAPFSMIANLLVGTGMGITFYYL FQEVPSLSERLPFTSVDRLPTFFGTAIFALEGIGVVMPLEN NMKTPTHFIGCPGVLNTGMFFVVSLYAFTGFFGYLKYGP DTKSSITLNLPQDEVLGQCVKLMIAVAIFFTYSLQFYVPM EIIWKNVRHWFGAKKNLAEYSIRIGIIILTLGTAIAIPNLGPF ISLVGAVCLSFLGLIFPAVIETVTYWDRPNGLGRFNWVLW KNLFLVSFGILGFLTGAYVSVLDIIHGEE
HaAAAP12 (*rna-XM_021331270.1*)	MVNEANGNAPAPQELETFLSQDEKKKEKVVSKYNMTKD LESADFDPFTERKLENPTSNMDTLTHLLKASLGTGILAMP KAFKCAGLVSGIFFTMLVALICTHCSYVLIKCAHVLYKKT KRTQMTFPEVGQTAFENGPEKFRPWGNAFRIFILVSLFLTY FGTCSVYTVIIAKNIVQVVAHHMGVEEDKVDIRLFILALL LPLIFMAWIRNLKYLAPVSMVANVFMGIGLGITFYYLVGT GSLQTDKINSMLIKAPVEWPEFFSLSIFAMEAIGVVMPLE

	NAMKTPKAMLGLCGVLNKGMSGVTLVYILLGFLGYLRYGEDVKDSITLNLENSEYPAQIVKISIAIAVYCTYGLQFFVCVEIMWNSIKDKFTKRPDLADYIMRTILVTACVLLAVAVPTIGPFMGVIGAFCFSILGLIAPALIEVITYWDIGFGPTKYLIWKNIIVVIFGLFALVFGTKDAIKEIIRVYSQ
HaAAAP13 (*rna-XM_021332347.1*)	MPKNILAEMGSQVGLTPRQNKKEAEEWARRQSGPMVNPSFEPDDFLPQSLSPVKDEKKRPGDKSIFLVNMKEKNIPEVEEYEPYDNRVVDHPTTNMETFLHLMKGSLGTGILAMPKAFSNAGYVVGSVGTVIIGVLCTYCIHILIDSCYVLCKRRKVPSLSYTAAAEAALLEGPDWCKACAPYAAHVVNAFLLVYQIGTCCVYVVFVSDNIYFVLTKHFGIEITVFQVMLCVLLPLILINWVRDLKYLAPFSAIANAVTIVSFGMILYYIFRDTPTLEGKVPAGKLANFPLFFGTVLFALEAIGVILPLENEMKTPRDFVGKFGVLNCSMISIIVLYVGMGLFGYLQYGDESAGSITLNLPNTEILANVVQCLLAFAIFITHGLACYVAIDILWNEYIGIRMLNNKHRLIWEYLLRTVIVLLTFGIAAAVPALELFISLFGALCLSALGLAFPALIQSCTYWYYVSRSERIRMIVKNAIVVLFGALGLVVGTWTSLERIIATFGTSVIETASNHTMAQNETLTP
HaAAAP14 (*rna-XM_021334515.1*)	MNDEKQPLLTGPSNSIDSIEQPVELVTAPVATSATSGKPKSDYHPAAERRLEHPTSNFDTLIHLLKGNIGTGILAMPDAFKNAGLIIGVFGTLLMGTICTHCMHMLIKCAHELCIRSQKPALGFSEVVEDSFALGPISVRPYAKTMKNIVSVFLVITQLGFCCVYFLFVATNLQDTMHFFHINLSVHAYLALLFPPILALAMVKNLKYLTPVSLVASIMTAWGLVITFYYILQDLPATNTVDAFSSWHQLPLYFGTAIYAFEGIGVILPLENNMKTPEDFGGWNGVLNTGMVIVAALYTAIGFFGYLKYGDHVLGSITLNLPNTLLAQSVRAVMAASIFLSYGLQFYVPMNIVWPYVKSKLTSEQALKHGEAVTRFVLISITFLAAALIPNLSGIISLVGAFSSSALALIFPPVIEIMTFWPDQLGRYQWMLYKDIFIIFFGFTGFVFGTFINVKNIFF
HaAAAP15 (*rna-XM_021336598.1*)	MPSILENIWPTSVISHHEDYTVGETTGGLSVLFTILCIVDLFGVFPVITLPKSVISCGIYGLPLVLSVFGLQLYTAVLLGKSWLLAQKITPDIKEKNRFPYAAIAELAFGPSAKTLVTFLIDAAVFGSGVPNFIIASQTLQIFWWKISGGEIGITYCVWMVILALLLCPVMWLGSPKDMKPLAISSVLIVATVAVTIWTCILRDYTSPTPVDNMVALEPQLEDFLIAYGILAFQFDIHPMLLTLQVDMKDSKKINAAVLGGFGVTGCMFAVTTILAAVRYGTALDNNILQSIPSSIPLYLVSLLVTLQLCLSSAVSHSALFQHFEDILKIPRDFCFRRCLLRSSVVALAVVLAESVPRFDLVMGLVGSTLTGPLMFICPPVFFLKLCYMKSKTPSNFFKLSEKRNGTQNGGFKNGDLSKSPLILDALQPRYRTFTEYAVEVTEDDDYSIRWYDIIFALIVMTLGIVATIVATYSSWSNSIAYATFSPPCLMNATAAARSFLEIPMAI

HaAAAP16 (*rna-XM_021337064.1*)	MNEGLFFSLFCFQRSPTPSLYTLDRDIKNYIHKRESHSYEY YPGTERTVYDYIEERNDMYNTNLIESTAHLIKGSLGAGAL SMHEAYMYGGLWTSLVVTIVIGITISYTMLMLIRSAQKM YRRLRISKLSYPDLAEAACATSPWRFVRKLSKPFRYLVDA CIIFEMCGTCCIYQIMIGSSIKQMFEGIDYEFAKRVMGIRLY ILISAPILLPLCQIRSLKFLAPFSMLADVFVALCVLTTLYYS LITLNANITDRYAWKNIHGFFRIAGICMYTTSGICVALPVE NNMKRPRYFPQAVRLGMFLTTIVVLTLTVTTGVFGYWAW GESCKSPITVHMPLNIFTTILQFLLIMMLSTSFAVQFWVPF RIIWHYLVRRYKRKQAIWERVHRLIMGLIVMAFTMLFPN VISVMIFLGQFFMGLVALVFPATIEIMVDWEEAYSHRARVR LCQHVKNFTLITMGLMLSLSTLYSVMDT
HaAAAP17 (*rna-XM_021337048.1*)	MELKPNEGRPGHTTHSTHVRHHTTPERRFVDRSAEGEYN YVAARNNAKPTNVLESTGHLIKGCLGGGILGIHEAYMKC GLWTSFFMTIMFGFYISYCVHILVSSAHKLYGRLHLATLS YPDVAEAALAIGPFPKLRKYSKMFRYAVDVIICIDLFGACC VYQIIIAKTIQQLTLSGQDSTSISYLRLFIIALLVPILLLCMIT TLKYLAPFSLVADFFIVACVIATIAYSLKIAPPIEDVPGWKN AMGFFEFSGVVVFSMEGVGVALPIENNMKEPKRFIVVLC AGMSVVVTFVLLVGFFGYWGFGEKCISPVTLNFPSEIFPTI LKVLIALMIFVXFALNYWAPFNLVWYYLAKRHDSSKHWI WERVYRAVFIIIITIISVIFPNIGNLMGLLGAFCLSNMGFIFP ALIDLLVVWENPGLGTLKWRLWKNVVILIAGFMLFVAGT YSNAKGLLFTTYE
HaAAAP18 (*rna-XM_021337340.1*)	MMEESNKSETVHLQPVGEGAGEGFKDSASEYYDPHEHR QLPKPTNNWETLVHLLKCSLGTGILAMPQAFARAGLVTGI LATIIVGALVTHCLHVLVRSQYAACKRLRVPLLTYPQSMS AALEAGPPPLRRFAGSLATTVDVFLVVYQLGICCVYIVFIA DNIKKMVDPYYTMAVEIHMLIILLPLIAFNLIPSLKLLAPFS AVANVLTFVGLGIVVYYLLTNKKSDAPLDLWGSPSTFPLF FGTILFALTAVGVVIALENNMATPKAFGAPCGVLNSGMVII VLLYVAVGALGYVFCVSECSDSVTLDLPQGALANSVIAM FAIAIFISYGLHCYVPVEVVWRGYLLPRLQRSGTPPARLRV AEYALRVVLCLLTFVLAVAVPRLGLFISLFGALCLSALGICF PALMEMCVSFPDKFGPGRVRLLKDVLLFIVGVVGMVAGT YTALLSIVRSFMPPDTEPLHA
HaAAAP19 (*rna-XM_021337359.1*)	MFDVFRLASPGGQPLQQPPEFDASFDPHKHRKVPHPTSY GETMTHMLKACFGAGMLAMPNAFARLGLIFGIVGIIFLG VFATYCIQILVLGQYQICKKWRVGYMSYPKSMRLAVQSG PPCLRWSAAIFENAVDGILLFWQIGVCAIFLVFVAENLKQV CLYAGHELSLRVLICCLYPILVIICLVKDLKLLAPLSTFANG CTIIGLVLIFFYLVEDDVQVDESHFALKSLTDIPIFIGIVLYA LEAVGVILALEYNMDNPADFTGLFGLFSIGMSIIVLIYTAL GVCGYLKYGMDSKASITLNLPQDQKKSHAAKMAFTLAV

	FSSFPLQNFVAWQILWQNYLEKKYKGTKKEKVADYGLRI<br>LCTTIPFVMALIAPALGPFIGLIGSFCLSMVAILFPAVVDISV<br>WYPDKYGPGKYKLFTDIFIIIFGIFCCCSGVYTSLLEMVET<br>LSK
HaAAAP20<br>(*rna-*<br>*XM_021337338.1*)	MFIYKFVGFGFDDTATDESTDAYDPHAHRIVAKPCTYGET<br>MMHMLKGCLGAGLLAMPNAVARMGIVLGALGIIFIGAFA<br>TYCIQILVLAQYQLCKRHRRGYMAYTKSIRVAILGGPPQL<br>HWSANCFANLVDFFLVFWQIGICAIYFVFVAENVKQVVEF<br>IGYEVTVRRVICYSYPPLLICSLVKDLKLLTPFSTISNACVL<br>VGLVLVFFYLVEDDVVLDEDKYKVKGLEDIPIFIGITLFAL<br>EAVGVILALEYNMEKPREFTGFCGLFSVGMMIILATYIAL<br>GVFGYLKYGNECKGSITLNLPQDEKKAQVAKLTFTIALFL<br>SYPLQNFVAWQIVWRKTHKKVSDKWKRTVDYALRVILAT<br>IPFGMAAAAPSLGAFMGLLGALCLTMVAILFPALMDLCV<br>YYPNRYGFMRWKLWSDIFIIVFGMVCCASGVYTSVLEMV<br>ETYDL
HaAAAP21<br>(*rna-*<br>*XM_021337468.1*)	MISRNLKELVEGDENLSDDGNPPLRVYILSMMLPCMVVC<br>MVSNLKYLAPFALIAVIYCVAVMVMTVWYACKNDKLMP<br>WDRPSYKNMMGFVKLIGMCIFVTETASVALPIENNMNTP<br>KKFHYVMVSAMPIVTLLMMTIGFCGYWNYGEIAFSPITV<br>HFPFDPFSIQLKVFLCILLYVMIAIYSYCAFDVEWFYFKRL<br>HKPANYWFWERVYRSLHVVVIDLIAYAAPTVSGTMSIIGC<br>MFTNPIVFFYPQLIEMLVCWDYPGLGRHNWRLVKCIFIMF<br>ISLVIMLGGTGLNVYRMIYDMYIKPRGRVVSNATTESNFF<br>DITGRLRSFNGDY
HaAAAP22<br>(*rna-*<br>*XM_021337469.1*)	MGLFEFMSVCTFVLEPVSYALPVENNMNEPKKFHNVVMI<br>AMSINTVAMITVGFVGFWHYGNFAVSPITIHLPFSPFALTL<br>KVGLCIILYVIYSMCFYVAFDIEWFYLKRHHQMSNYWFW<br>ERLYRTIHVIILIMVSYCLPNVTRLMGIIGALCTSPAVFIYPY<br>FIELLLDWEHPGLGRCKWRLLRFIVMLTFGLVLFVAGTYY<br>NGLGMYLELENRPKANISFDPENQMPELERKEINFEIGTE<br>LSVDPKKRLNLTDEGKHNYDLNVALPYEVPYNLFDQNLN<br>ANYSTKKANIVFDHTM
HaAAAP23<br>(*rna-*<br>*XM_021339027.1*)	MSKKVLEPRAKAGVSSENVSETTPLVGKKGEGDEEGRGL<br>TTQQTAILIAGEMAGSGVLALPRALSKTGWFGVPLLLLVA<br>LVAAFSGKRLGDCWSILEARDPEMRTRKRNPYAIIVEQAL<br>GKPWSVVVSMAMIVTLFGASVVYLLLAAQIIEAVLLSLVP<br>TLTICTWYLIVVGAMTPLLFFGTPKDFHLMGILAFGSSLVA<br>CILYFIEMMNEVSPFTYRYGIHGFMDFFLATGTMMFAFGG<br>ASTFPTIQNDMADKTKFGKSVQYGFLAVLILYLLVAIAGY<br>AVYGERVLPNVALSMSATPLTLAANILMAVHLLSAFIIIINP<br>VCQEVEELYNVPRDSTGWRMIVRVSIMLGILFIGESIPRFY<br>TLLALVGATTIALLTYILPSVCYLKLIGQPAGEGQTPVEVP<br>SWMKMVCYEVIALGVVGCIAATISAVSAIFSTSMAVPCYL

HaAAAP24 (*rna-XM_021341781.1*)	MRFVEKIAKQAREPDSAASSVRSVDSYEIFGSACSDFTDC<br>EYCEDRRKRYKKYNKYHSPSITSHSYYSAMSRAEAEPLL<br>QGAVTPTYRAVSVESIDSLGPAELSSDAVLETFNKTFEKTF<br>KPDTKKQSSLVTIFSVWNTIMGSSLLTMAWGVERAGLPA<br>ALVLVAFMAALCLYTAYVLLRVNKHHGSDSCEVPALCRT<br>LLGQWAEVVAHIFSLLVLLGANIVYWILITNFLYFTVNYF<br>VDLNSSNETIYNTSLLCPRHLNESLLIAEPEADSAYWGLH<br>TTVPIYVALIVFPLLNFKNVSFFTKFNSLGTLSVAYLLIFVL<br>VKGYAWGIHIGELFTQTHAIRNAAVLSGMLALSFYIHNIII<br>TIMSNNARQDKNGRDLTIAFLLVTVTYTLVGAVFYICFPL<br>AKSCIEDNLLNNFEMHDVMTAVARMLLLFQVVTVYPLVA<br>FMLRSEAILLLPFKETRGLTIIINISIMAMCILVACFCPSIGTII<br>RYTGAVSGLVHIFALPSLLQIRSLQFRGKLTWWKTVFYCSI<br>MVFGAVNLLMQFFIDE
HaAAAP25 (*rna-XM_021342544.1*)	MINLGKFKLPPLRNVLDVTMQTVRQQIPEKPGAPPRPPQN<br>VRFANLDNMGESCELSTMNETTSPSYQSTNPTNPFLSGAL<br>QAEDSFTSYQNTYPQQDGAPRTQSMQSVDFYASSEEGGG<br>FEEGGGPPGAKINEYQAAWNVTNAIQGMFVVSLPFAVLQ<br>GGYWAIAAMIGIAHICCYTGKILVECLYEDDPVSGQRVRV<br>RDSYVSIAKECFGRKYGARIVNLAQIIELLMTCILYVVVC<br>GDLMIGTFPDGAIDARSWMMLTGIFLLPLAFLKSLKSVSM<br>LSFWCTMSHLIINAIVLGYCILNIGDWGWSKVKWTLDFE<br>NFPISLGVIVFSYTSQIFLPTLEGNMEDRSKFEWMLDWSHI<br>AAAAFKSIFGYLCFLTFQNDTQQVITNNLRSAGFKGLVNF<br>FLVIKAVLSYPLPYYAACDLLERALFRGKPKTIFPVIYALD<br>GELKVWGLAWRLGVIMFTILMAIFIPHFAILMGFIGSFTGT<br>MLSFIWPAYFHLKLKGNTLESTRIAYDYFIIALGVLFGVIG<br>MYDSGSALIKAFKIGLPF
HaAAAP26 (*rna-XM_021343518.1*)	MVDKKMQHEINITHISGSVVSDIHEINSLSGTLPETKKQEA<br>HNGGHHVVTHPTSYADTMLHLFRGNIGTGLLAMGDAFK<br>NGGIVFSPIVTALLGIICVHAQHLLLNCSEEMHRKSKRDR<br>APGFAETVYLVFASGPVSCRSMATTMKILVNGFLCVTQLG<br>FCCVYIVFIANNVKMICDQYNIHISLSIHMLFVVLPIMLAC<br>MVRNLKYLTPFSTIANIMMAFGVAVVIYAATQDLPAVETR<br>VYMASWQQLPLYFGTAIYAFEGIGLVLPLKNEMSNPESFQ<br>KPLGVLNVGMVVVGSIFITVGFLGYLKWGDEVLGSLTLN<br>LQPGEVLSNVVQSLIALAILFTYPLQFYVPIAITWPAIEKK<br>YGATHPVAKELGYRALLVILTFILAESIPELGLFISLVGAVS<br>STALALMFPPLLELVSTSQKPGGIPTHILIKNCFIILLGLFIFS<br>TGTYESVASIVRAFQV

Table S2. TPM values of putative AAAP transporter genes in BPH at 15 different developmental stages.

Gene	egg24h	egg48h	Egg5D	1st24h	1st48h	2nd24h	2nd48h	3rd24h	3rd48h	4th24h	4th48h	5th24h	5th48h	A24h	A72h
NlAAAP01	4.12	0.24	2.36	36.75	37.39	29.38	39.39	33.54	30.57	32.18	32.92	32.60	35.34	54.68	44.89
NlAAAP02	0.62	0.42	16.33	30.62	26.59	25.55	21.34	22.02	20.13	18.55	14.00	18.63	13.17	18.54	13.98
NlAAAP03	5.27	5.99	4.65	7.95	12.73	13.23	17.94	16.39	17.04	15.49	15.41	16.18	18.97	20.36	12.14
NlAAAP04	13.09	8.41	12.01	7.45	7.11	8.32	6.95	8.12	8.24	7.39	6.96	6.54	8.12	8.73	10.00
NlAAAP05	2.48	1.30	15.63	12.82	15.20	16.90	9.88	16.62	17.16	8.23	12.14	9.21	11.58	6.61	5.50
NlAAAP06	2.69	3.05	13.14	13.34	10.01	9.02	8.20	6.95	8.24	7.90	9.43	12.15	11.97	14.20	17.08
NlAAAP07	40.90	36.28	16.00	26.77	39.94	22.17	43.77	27.89	46.15	34.97	21.82	32.64	48.92	145.48	115.50
NlAAAP08	0.37	0.47	5.30	9.13	9.23	8.65	9.16	8.20	7.90	14.97	10.74	11.43	10.89	8.39	7.20
NlAAAP09	6.26	9.37	24.64	13.67	11.81	12.77	11.57	19.67	7.17	28.77	37.17	29.61	24.89	14.39	15.31
NlAAAP10	5.14	1.41	17.60	21.66	27.31	17.76	21.99	14.59	15.27	12.55	8.39	11.92	12.24	18.44	12.67
NlAAAP11	1.47	0.30	3.53	5.95	9.30	5.07	12.98	4.74	7.15	2.64	2.24	3.42	2.38	4.75	2.90
NlAAAP12	24.34	7.09	14.54	62.70	88.38	109.02	100.15	102.77	99.55	96.26	84.80	117.09	114.71	103.03	93.25
NlAAAP13	30.11	25.18	7.09	48.46	45.23	39.11	43.84	40.05	23.25	37.52	23.36	41.29	40.44	39.61	39.74
NlAAAP14	1.48	1.74	5.27	10.48	6.62	5.50	5.69	7.73	4.13	12.16	7.94	9.50	10.46	6.72	3.71
NlAAAP15	1.51	0.96	8.29	22.56	20.05	19.48	13.90	28.63	15.08	24.41	12.97	19.91	26.32	26.25	15.45
NlAAAP16	0.06	0.14	0.03	0.05	0.00	0.03	0.08	0.03	0.18	7.98	13.35	12.75	4.50	0.00	0.00
NlAAAP17	5.12	5.19	3.39	5.34	4.62	3.70	3.97	4.12	4.02	5.89	4.73	7.85	6.29	6.72	6.84
NlAAAP18	6.44	4.99	15.29	8.18	10.30	8.58	10.56	8.27	10.46	10.64	7.89	10.64	14.64	20.03	23.31
NlAAAP19	5.33	6.47	6.34	5.04	5.34	6.08	4.14	6.30	5.03	5.87	5.28	5.20	8.60	10.27	10.13
NlAAAP20	3.44	2.87	7.86	6.71	6.07	5.56	5.65	7.94	4.49	5.49	3.21	4.23	3.44	1.86	1.51
NlAAAP21	5.28	3.99	5.79	12.81	8.92	7.78	6.42	7.41	6.19	6.19	5.16	7.00	6.85	3.31	2.91

Table S3. TPM values of putative AAAP transporter genes in BPH at six different tissues.

Gene	Head	Salivary Gland	Integument	Gut	Fat Body	Ovary
NlAAAP01	4.86	0.09	49.08	76.12	39.64	1.86
NlAAAP02	16.21	6.02	3.37	352.84	3.63	6.19
NlAAAP03	3.73	2.52	3.14	91.03	0.44	4.96
NlAAAP04	3.33	5.00	9.76	7.93	9.67	13.80
NlAAAP05	8.20	1.22	3.39	19.39	3.74	7.97
NlAAAP06	0.31	0.87	3.73	0.93	6.58	17.37
NlAAAP07	12.17	10.18	83.48	6.20	29.57	70.85
NlAAAP08	2.10	0.57	1.11	100.51	1.07	1.68
NlAAAP09	26.78	5.07	12.78	93.08	22.02	4.79
NlAAAP10	2.12	0.19	1.20	0.39	0.06	0.31
NlAAAP11	10.41	1.60	3.49	0.55	0.68	2.22
NlAAAP12	42.72	9.02	87.94	1362.75	28.37	53.34
NlAAAP13	15.41	7.34	8.12	211.23	7.95	39.97
NlAAAP14	8.02	4.39	7.48	2.39	1.02	1.32
NlAAAP15	18.30	5.23	13.26	44.71	15.57	18.75
NlAAAP16	0.00	0.00	0.00	0.00	0.08	0.05
NlAAAP17	3.01	0.63	6.49	4.68	5.45	10.81
NlAAAP18	8.34	8.47	15.74	9.35	17.34	8.85
NlAAAP19	0.94	1.73	5.61	6.72	3.07	7.96
NlAAAP20	11.91	0.56	0.51	0.98	1.41	1.08
NlAAAP21	1.39	0.35	1.93	0.72	2.67	2.47

Table S4. The primers used for qPCR and RNAi experiments in this study.

Primer	Primer sequence (5'-3')	Purpose
NlAAAP01-F	ATGGAAGTGGTGTGGTCTGC	Quatitative real-time PCR
NlAAAP01-R	GAACGGCTATGCTGCTCAGA	Quatitative real-time PCR
NlAAAP02-F	GAATTGGCTCCAGCATCAACG	Quatitative real-time PCR
NlAAAP02-R	GCGCCCAAACTGCTTTTGAT	Quatitative real-time PCR
NlAAAP06-F	CCTCGATGGTGATCGTAGCC	Quatitative real-time PCR
NlAAAP06-R	CCAATAAATCGCGCTGAGGC	Quatitative real-time PCR
NlAAAP07-F	TGGGAACAGGAAACGTGAAA	Quatitative real-time PCR
NlAAAP07-R	AAGTAGGCAGCGAACAGTCC	Quatitative real-time PCR
NlAAAP09-F	ACTACGTTACGTTCCGGTCG	Quatitative real-time PCR
NlAAAP09-R	TACCTGGGTTGGCGTCTCTA	Quatitative real-time PCR
NlAAAP12-F	CGTTGGGTCCGCACAAGA	Quatitative real-time PCR
NlAAAP12-R	TAACCAAGGCAGGGTTGTCC	Quatitative real-time PCR
NlAAAP13-F	TGAGATATCACGAGAATAAGGGTCG	Quatitative real-time PCR
NlAAAP13-R	CTCTTCGGCCTCCTTACTGG	Quatitative real-time PCR
NlAAAP16-F	AGCCATCACAATTGCTATAAGTTCT	Quatitative real-time PCR
NlAAAP16-R	TTCTTCGTCAATTCGGGACCA	Quatitative real-time PCR
NlAAAP18-F	CTGAAGCATCCACCTCAGCA	Quatitative real-time PCR
NlAAAP18-R	GTTCTGGATGGTGGGGAAGG	Quatitative real-time PCR
NlAAAP20-F	GGCGCACCTGTTCAACTTTC	Quatitative real-time PCR
NlAAAP20-F	CCGTTGTTGGTGCTTTCCAC	Quatitative real-time PCR
ds*NlAAAP07*-F	TTGGGAACAGGAAACGTGAA	synthesis of dsRNA for *NlAAAP07*
ds*NlAAAP07*-R	GCTAATCAGCACCAGTGGGA	synthesis of dsRNA for *NlAAAP07*
T7- *dsNlAAAP07-F*	GGATCCTAATACGACTCACTATAGG TTGGGAACAGGAAACGTGAA	synthesis of dsRNA for *NlAAAP07*
T7- *dsNlAAAP07-R*	GGATCCTAATACGACTCACTATAGG GCTAATCAGCACCAGTGGGA	synthesis of dsRNA for *NlAAAP07*
dsGFP-F	AAGGGCGAGGAGCTGTTCACCG	synthesis of dsRNA for *GFP*
dsGFP-R	CAGCAGGACCATGTGATCGCGC	synthesis of dsRNA for *GFP*
T7- *dsGFP-F*	GGATCCTAATACGACTCACTATAGG AAGGGCGAGGAGCTGTTCACCG	synthesis of dsRNA for *GFP*
T7- *dsGFP-R*	GGATCCTAATACGACTCACTATAGG CAGCAGGACCATGTGATCGCGC	synthesis of dsRNA for *GFP*
